# Deeper below the surface—transcriptional changes in selected genes of *Clostridium beijerinckii* in response to butanol shock

**DOI:** 10.1002/mbo3.1146

**Published:** 2020-12-14

**Authors:** Petra Patakova, Jan Kolek, Katerina Jureckova, Barbora Branska, Karel Sedlar, Maryna Vasylkivska, Ivo Provaznik

**Affiliations:** ^1^ Department of Biotechnology University of Chemistry and Technology Prague Prague Czech Republic; ^2^ Department of Biomedical Engineering Brno University of Technology Brno Czech Republic

**Keywords:** ABE fermentation, butanol shock, *Clostridium beijerinckii*, transcriptome analysis

## Abstract

The main bottleneck in the return of industrial butanol production from renewable feedstock through acetone–butanol–ethanol (ABE) fermentation by clostridia, such as *Clostridium beijerinckii*, is the low final butanol concentration. The problem is caused by the high toxicity of butanol to the production cells, and therefore, understanding the mechanisms by which clostridia react to butanol shock is of key importance. Detailed analyses of transcriptome data that were obtained after butanol shock and their comparison with data from standard ABE fermentation have resulted in new findings, while confirmed expected population responses. Although butanol shock resulted in upregulation of heat shock protein genes, their regulation is different than was assumed based on standard ABE fermentation transcriptome data. While glucose uptake, glycolysis, and acidogenesis genes were downregulated after butanol shock, solventogenesis genes were upregulated. Cyclopropanation of fatty acids and formation of plasmalogens seem to be significant processes involved in cell membrane stabilization in the presence of butanol. Surprisingly, one of the three identified Agr quorum‐sensing system genes was upregulated. Upregulation of several putative butanol efflux pumps was described after butanol addition and a large putative polyketide gene cluster was found, the transcription of which seemed to depend on the concentration of butanol.

## Graphical abstract

Detailed analyses of transcriptome data obtained after butanol shock and their comparison with data from standard acetone–butanol–ethanol (ABE) fermentation revealed new findings and confirmed the expected population response. Although butanol shock resulted in upregulation of heat shock protein genes, their regulation is probably different than was assumed based on transcriptome data from standard ABE fermentation. While glucose uptake, glycolysis, and acidogenesis genes were downregulated after butanol shock, solventogenesis genes were upregulated.

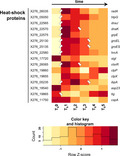

## 1 INTRODUCTION

Clostridia represent ancient, anaerobic, and the first bacteria on Earth able to develop a successful survival strategy (Martin & Sousa, 2016). This is exceptional considering all massive extinction events (including the Great Oxygenation Event) in which most of the known species died out. Yet, their fascinating adaptive mechanisms enabling them to cope with hostile environments have not been fully described and understood.

*Clostridium beijerinckii* is a non‐pathogenic, non‐toxigenic, solventogenic clostridial species that belongs to Cluster I (*sensu stricto*) of the *Clostridium* genus but is distinct from its more familiar relative *Clostridium acetobutylicum*. According to Cruz‐Morales et al. (2019), the *C. beijerinckii* species may be sorted into subgroup 7 of Cluster I with *C. butyricum*. The *C. beijerinckii* species is capable of producing butanol and hydrogen via the acetone–butanol–ethanol (ABE) fermentation pathway, which is the main method for obtaining energy from the bacteria (Lee et al., 2008). In the same way, butanol and hydrogen are valuable chemicals, usable as fuels or starting blocks for different chemical syntheses. The bacteria can produce them from a spectrum of waste materials such as wheat straw combined with chicken feathers after pre‐treatment (Branska et al., 2020), distillers dry grain solubles (Ezeji et al., 2007), brewery liquid wastes, apple pomace (Maiti et al., 2016), and others. However, the industrial use of this technology is not cost‐effective, mainly because of the low concentration of products. Thus, resolving this bottleneck remains an issue to make the technology previously used at the industrial scale more competitive with chemical synthesis (Moon et al., 2016).

Butanol toxicity seems to be a major obstacle that prevents the attainment of a high butanol concentration in the culture medium after fermentation. Different types of butanol stress responses studied in different microorganisms, including cell membrane/wall modification, stress protein formation, transport, quorum sensing, efflux stimulation, accumulation of protective compounds in cells, chemotaxis/motility, and sporulation were reviewed by Patakova et al. (2018). In solventogenic clostridia, butanol shock response was described mainly in *C. acetobutylicum* (Alsaker et al., 2010; Tomas et al., 2004).

For the current study, the strain *C. beijerinckii* NRRL B‐598, originally *C. pasteurianum* (Sedlar et al., 2015), reclassified in 2017 (Sedlar et al., 2017) was selected because the course of the standard ABE fermentation and main life cycle events have been described at the transcriptional level (Patakova et al., 2019; Sedlar et al., 2018; Vasylkivska et al., 2019) in this strain and these results can be used for comparison. Clusters of genes differentially regulated after butanol addition have been described recently (Sedlar et al., 2019). The main goal of this article is to reveal surprising, unexpected transcriptional changes with the expected ones. Our attention is focused on central glucose metabolism, sporulation, Agr quorum sensing, and different types of stress response. The core of this work consists of the analysis of transcriptomic data of the relevant selected genes.

## 2 MATERIALS AND METHODS

### 2.1 Culture and maintenance

*Clostridium beijerinckii* NRRL B‐598 from ARS/NRRL culture collection (previously *C. pasteurianum* NRRL B‐598; Sedlar et al., 2017) was used in the study.

### 2.2 Growth medium and culture conditions

Culture medium, inoculation, and culture conditions were described in the preceding article (Sedlar et al., 2019). For batch bioreactor cultivation of *C. beijerinckii* NRRL B‐598 with butanol shock, pure HPLC‐grade butanol (Sigma‐Aldrich) was added to the cultivation medium at the 6th hour of cultivation under sterile and anaerobic conditions, to concentrations of 4.17 and 4.34 g/L in the first and second reactors, respectively. Throughout cultivation, samples of culture broth were taken for flow cytometry analysis, OD measurement, HPLC analysis, and subsequent total RNA isolation. Sampling for total RNA isolation was done at the 6th (directly before butanol addition, time point Tb0), 6.5th (Tb1), 7th (Tb2), 8th (Tb3), 10th (Tb4), and 12th (Tb5) hours of cultivation.

### 2.3 Flow cytometry combined with the fluorescent staining

Flow cytometry (BD Accuri C6 cytometer) along with fluorescent staining was used for evaluating the physiological state of the population. Double staining with propidium iodide and carboxyfluorescein diacetate was applied to the cell suspension for the determination of live/dead cells and the proportion of spores. In Table A1, the sum of CFDA‐stained and PI+CFDA (double) stained cells was taken as “active” cells, while PI‐stained cells were taken as “non‐active” cells; the number of spores reflects the number of mature spores released from mother cells (spores were recognized based on their fluorescence pattern and uniform light scatter signal). The analysis was done according to the method published by Branska et al. (2018).

### 2.4 Analytical methods

Cell growth was quantified using a spectrophotometer with absorbance at 600 nm (OD_600_) (Varian Cary 50 UV‐VIS spectrophotometer, Varian). The concentrations of glucose and metabolites produced by *C. beijerinckii* NRRL B‐598 were analyzed using HPLC. Sample preparation and conditions of the analysis were the same as described in Patakova et al. (2019) and Vasylkivska et al. (2019).

### 2.5 RNA isolation and sequencing

Samples for total RNA isolation consisting of 3 ml of culture broth at a cell concentration of OD_600_ = 1 were centrifuged, washed with sterile distilled water, frozen, and stored at −70°C. Total RNA was isolated using a High Pure RNA Isolation Kit (Roche) according to the manufacturer's instructions. MICROBExpress™ Bacterial mRNA Enrichment Kit (Ambion) was used for ribodepletion. Isolated total RNA and RNA after ribodepletion were stored in TE Buffer at −70°C. Quality, integrity, and concentration of isolated total RNA as well as RNA after ribodepletion were measured on a DS‐11 FX +Spectrophotometer (DeNovix) and Agilent 2100 Bioanalyzer (Agilent) using the Agilent RNA 6000 Nano Kit (Agilent). Library construction and sequencing of acquired RNA samples were carried out in the CEITEC Genomics core facility (Brno, Czechia) on Illumina NextSeq, single‐end, 75 bp.

### 2.6 Bioinformatics analysis

Raw RNA‐Seq data were preprocessed within our previous studies (Sedlar et al., 2018, 2019). Additionally, mapped reads for each sample were counted using the featureCounts function from the Rsubread package (Liao et al., 2014) in R/Bioconductor while each read was weighted by the number of mapped genomic features. RPKM values of read counts were evaluated using the edgeR package (Robinson et al., 2010) in R/Bioconductor, and mean values of RPKM were estimated for each time point of both cultivations. Data normalization was done in the DESeq2 package (Love et al., 2014) in R/Bioconductor separately for standard cultivation data and data from cultivation with butanol shock. Differential expression analysis among adjacent time points was followed by showing normalized average expression in heatmaps using a Z‐score of normalized mapped read counts. Heatmaps were generated using the gplots and RColorBrewer packages in R. Analysis of putative operon structures was performed using the Genome2D tool (available at http://genome2d.molgenrug.nl/index.html; Baerends et al., 2004). Furthermore, we used RNA‐Seq data for correlation analysis of expression profiles. Read counts from both cultivations were normalized together using DESeq, and mean values of normalized read counts for each time point were used for the analysis. A correlation matrix with Pearson correlation coefficients was computed for each putative operon structure.

## 3 RESULTS AND DISCUSSION

The main goal of the study was to capture changes elicited by the addition of approx. 0.5% v/v of butanol to the *C. beijerinckii* NRRL B‐598 culture at the 6th hour of batch fermentation and compare them to data obtained during standard ABE fermentation without interventions. During the first 6 h of fermentation, both shocked and standard cultures reached approximately the same point in ABE fermentation, which may be described as a point shortly after metabolic switch from acidogenic to solventogenic fermentation; there were already low concentrations of butanol (0.3 and 0.2 g/L for both fermentations). Regarding the physiological state of both cultures at the 6th h, the ratio of viable versus non‐viable cells, determined using flow cytometry along with double fluorescent staining with propidium iodide (PI) and carboxyfluorescein diacetate (CFDA), was comparable (approximately 75% live cells). From this point, the fermentation courses differed, sampling points were different for both of them and it is not possible to mutually compare the results reached for individual samples in both fermentations. Instead of comparisons of individual samples, it is necessary to compare overall changes during both experiments. More detailed data describing growth, production, and physiological states of standard and shocked cultures are summarized in Table A1 and were published in Patakova et al. (2019), Sedlar et al. (2019), and Vasylkivska et al. (2019).

**Figure 1:**
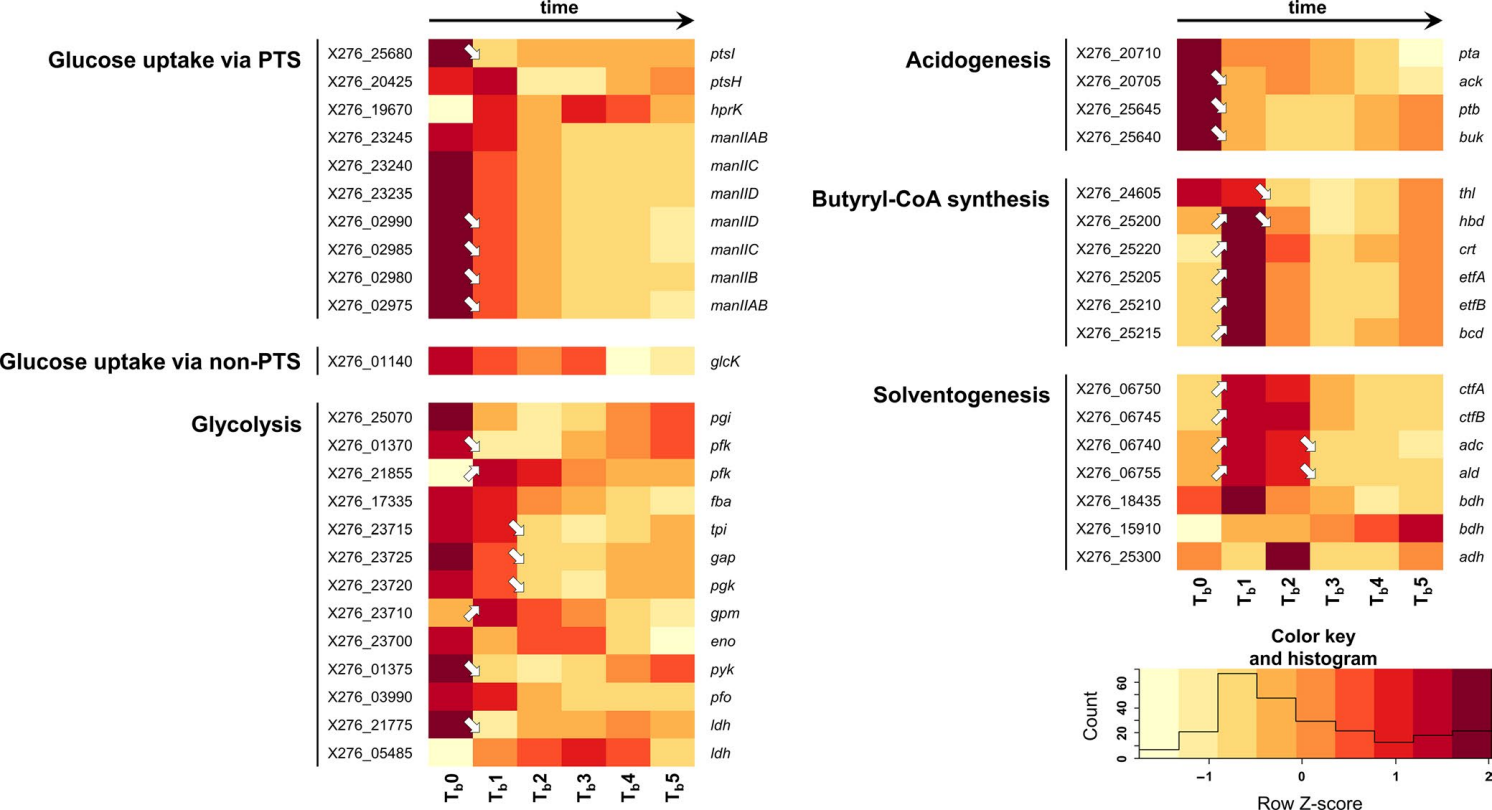
Heatmap of central metabolism genes during shocked ABE fermentation (arrows ↗ and ↘ indicate statistically significant (*p*‐adj <0.001, Benjamini–Hochberg correction) upregulation and downregulation of related genes transcription; if there is no arrow in the figure, transcription was not changed significantly). For genes description, RPKM values of standard and shocked fermentation, and heatmap of standard fermentation, see Table A2

**Figure 2:**
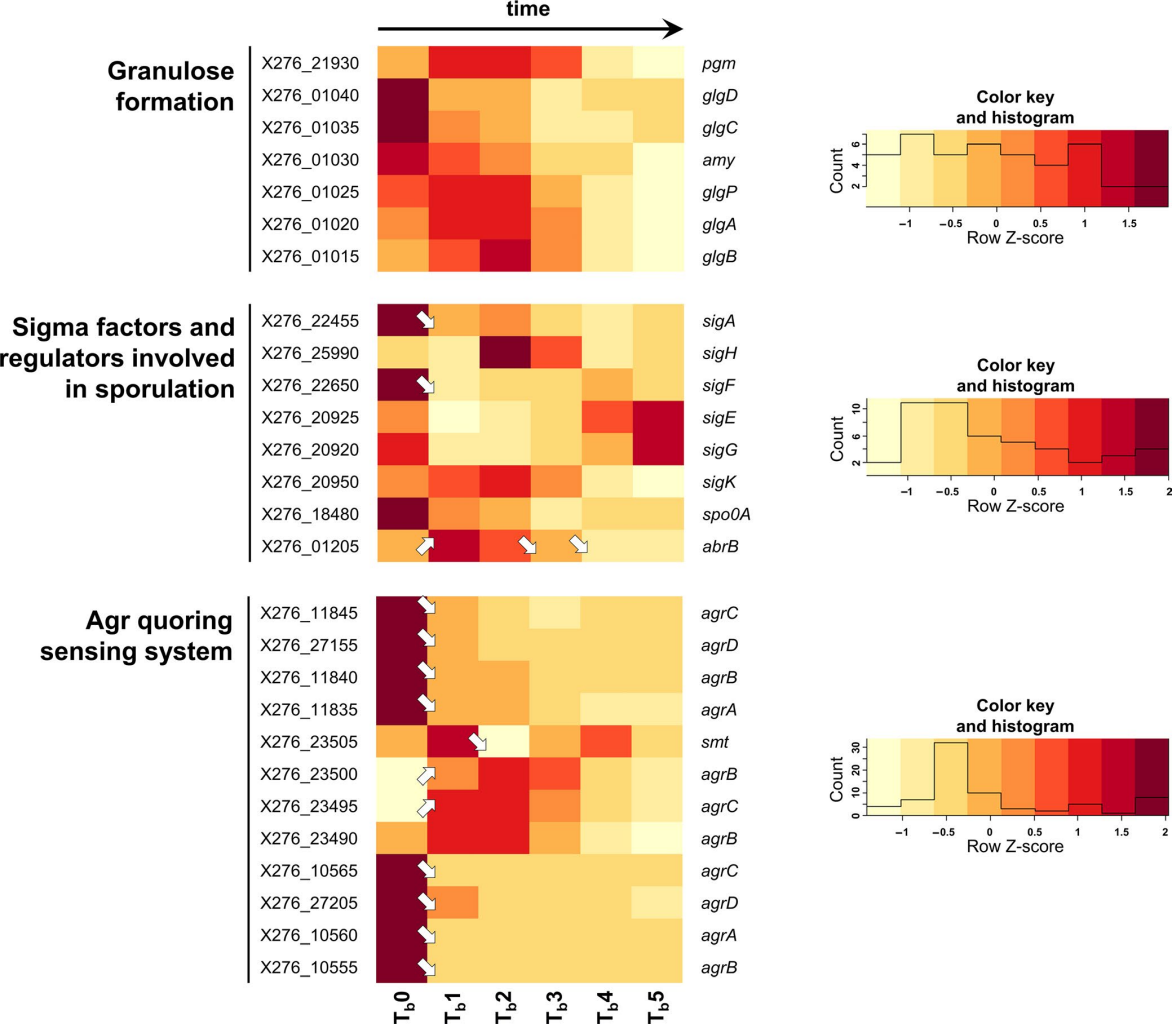
Heatmap of selected granulose formation, sigma factors, and Agr quorum sensing genes during shocked ABE fermentation. (arrows ↗ or ↘ indicate statistically significant (*p*‐adj <0.001, Benjamini–Hochberg correction) upregulation and downregulation of related genes transcription; if there is no arrow in the figure, transcription was not changed significantly). For genes description and RPKM values of standard and shocked fermentation and heatmap of standard fermentation, see Table A3

**Figure 3:**
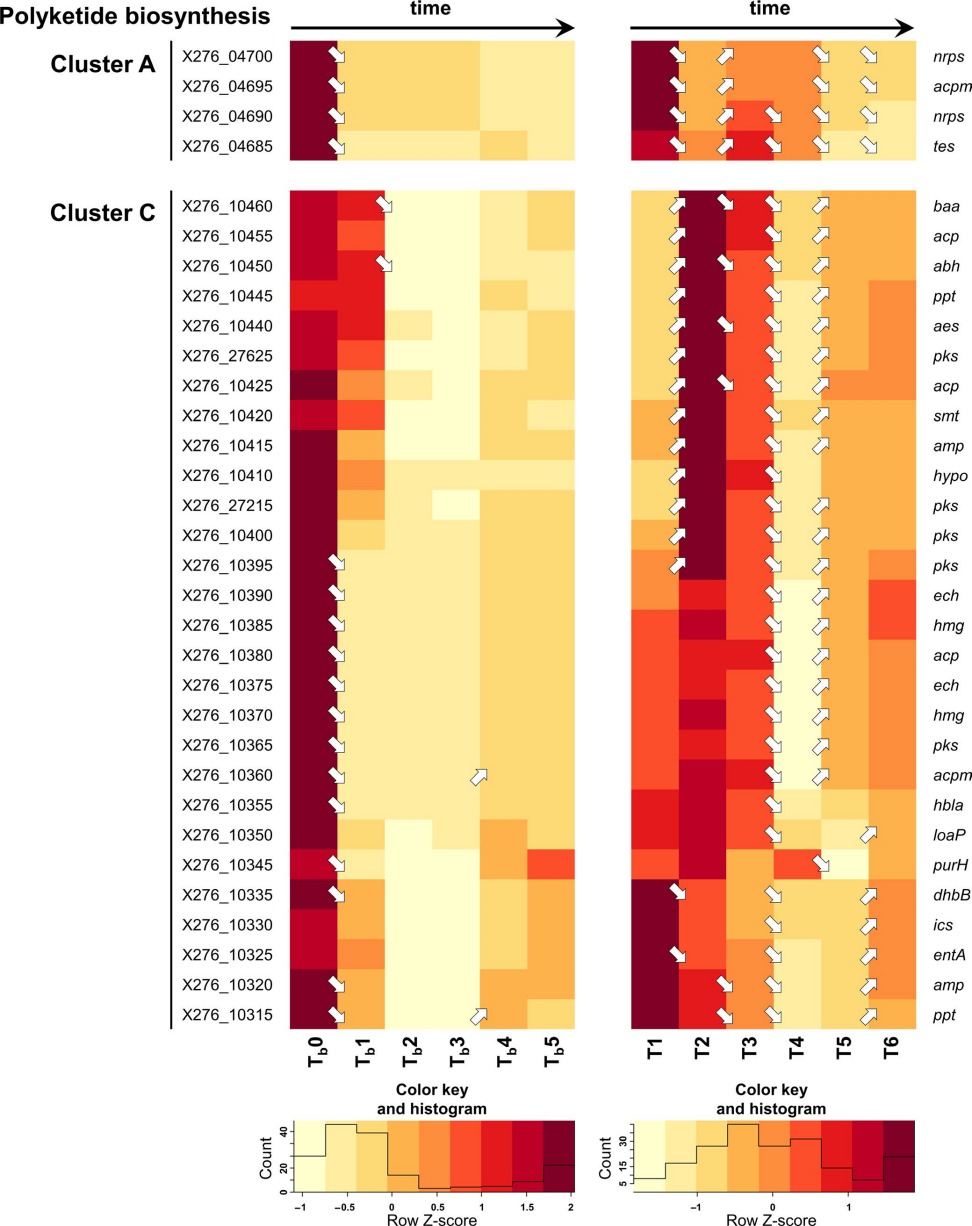
Heatmap of putative polyketide synthesis genes for shocked and standard ABE fermentations (arrows ↗ or ↘ indicate statistically significant (*p*‐adj <0.001, Benjamini–Hochberg correction) upregulation and downregulation of related genes transcription; if there is no arrow in the figure, transcription was not changed significantly). For genes description and RPKM values of standard and shocked fermentation and heatmap of standard fermentation, see Table A4

**Figure 4:**
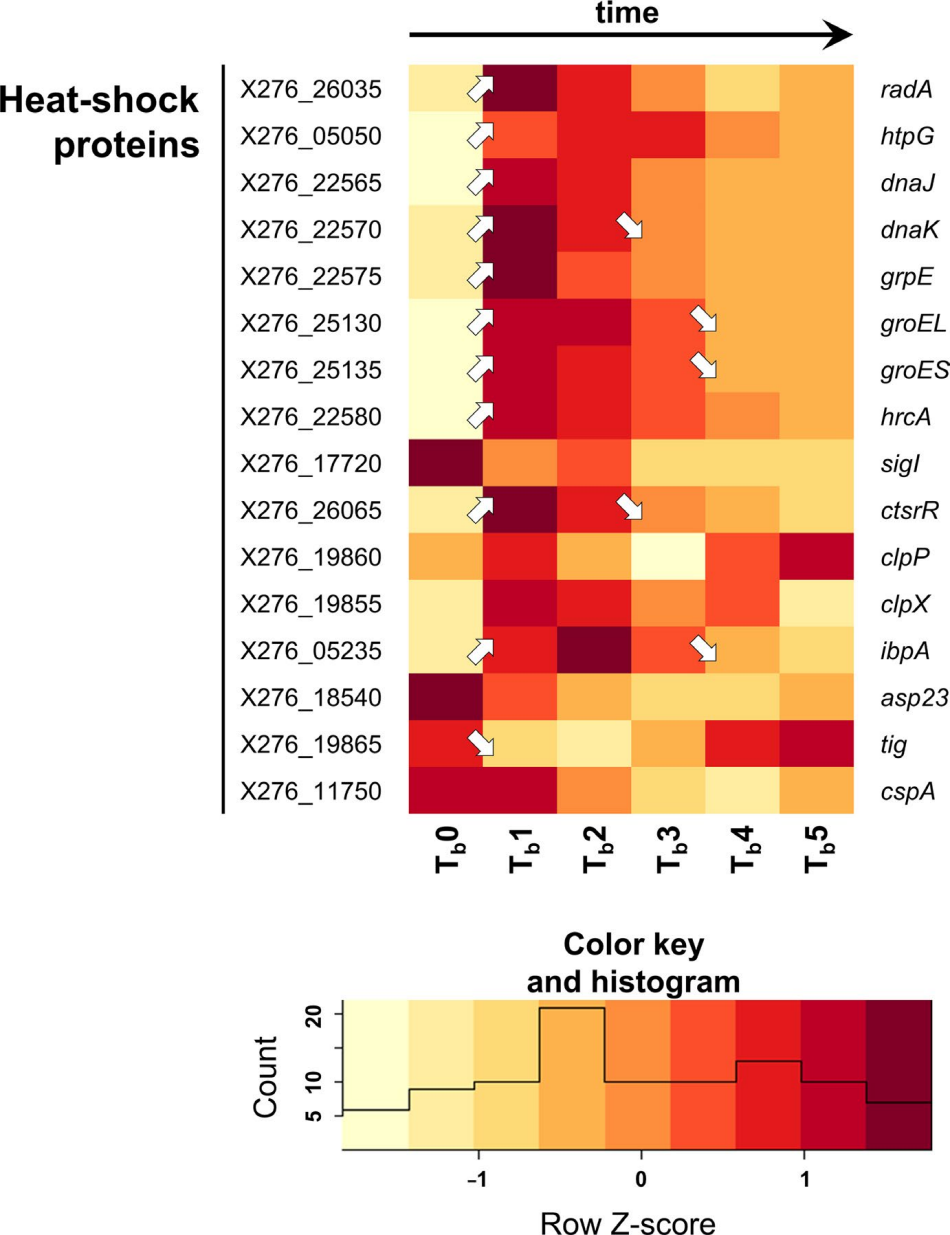
Heatmap of selected heat shock protein genes during shocked ABE fermentation (arrows ↗ and ↘ indicate statistically significant (*p*‐adj <0.001, Benjamini–Hochberg correction) upregulation and downregulation of related genes transcription; if there is no arrow in the figure, transcription was not changed significantly). For genes description and RPKM values of standard and shocked fermentation and heatmap of standard fermentation, see Table A5

**Figure 5:**
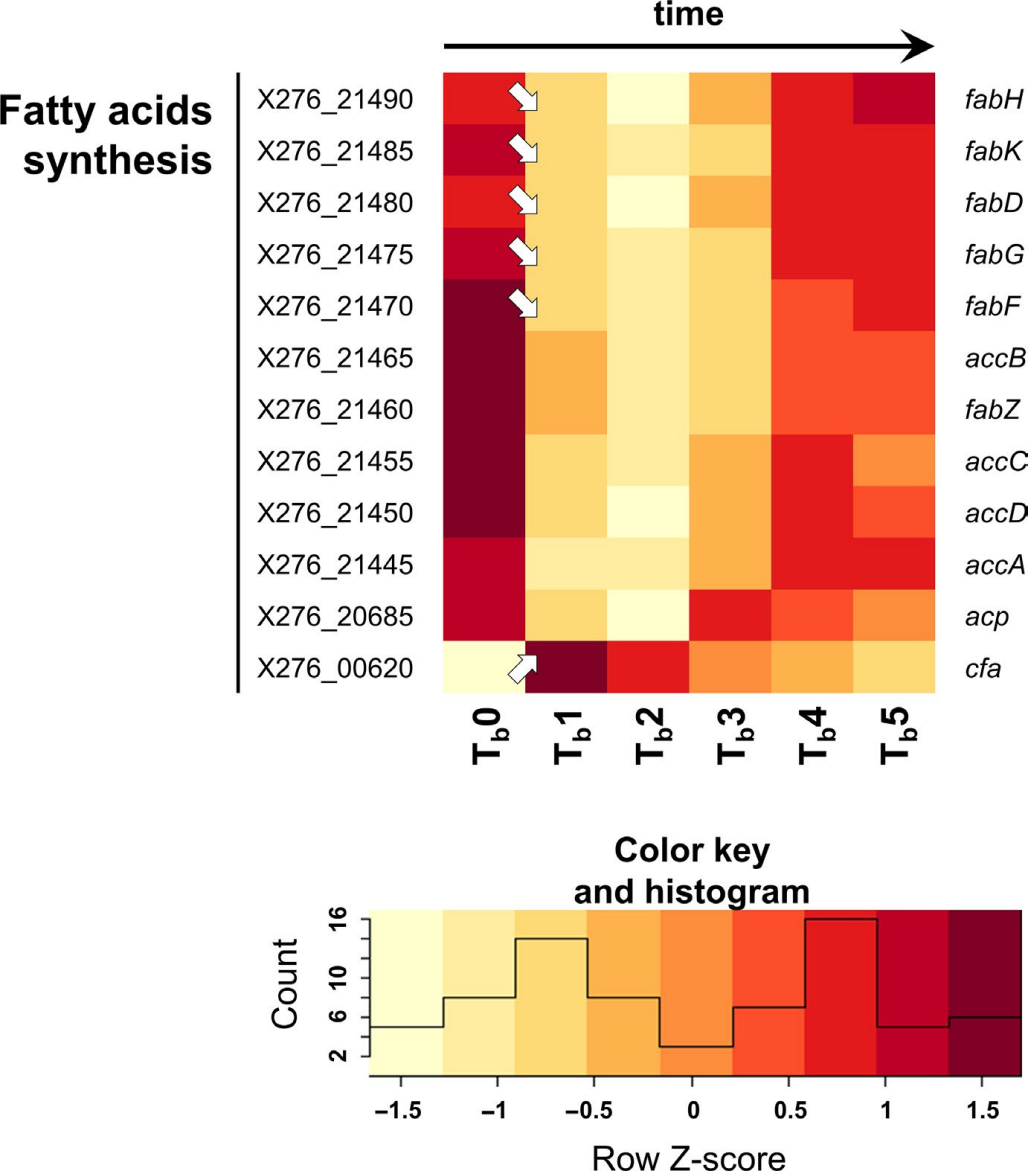
Heatmap of selected fatty acid synthesis genes during shocked ABE fermentation (arrows ↗ and ↘ indicate statistically significant (*p*‐adj <0.001, Benjamini–Hochberg correction) upregulation and downregulation of related genes transcription; if there is no arrow in the figure, transcription was not changed significantly). For genes description, putative operon organization, RPKM values of standard and shocked fermentation, and heatmap of standard fermentation see Table A6

**Figure 6:**
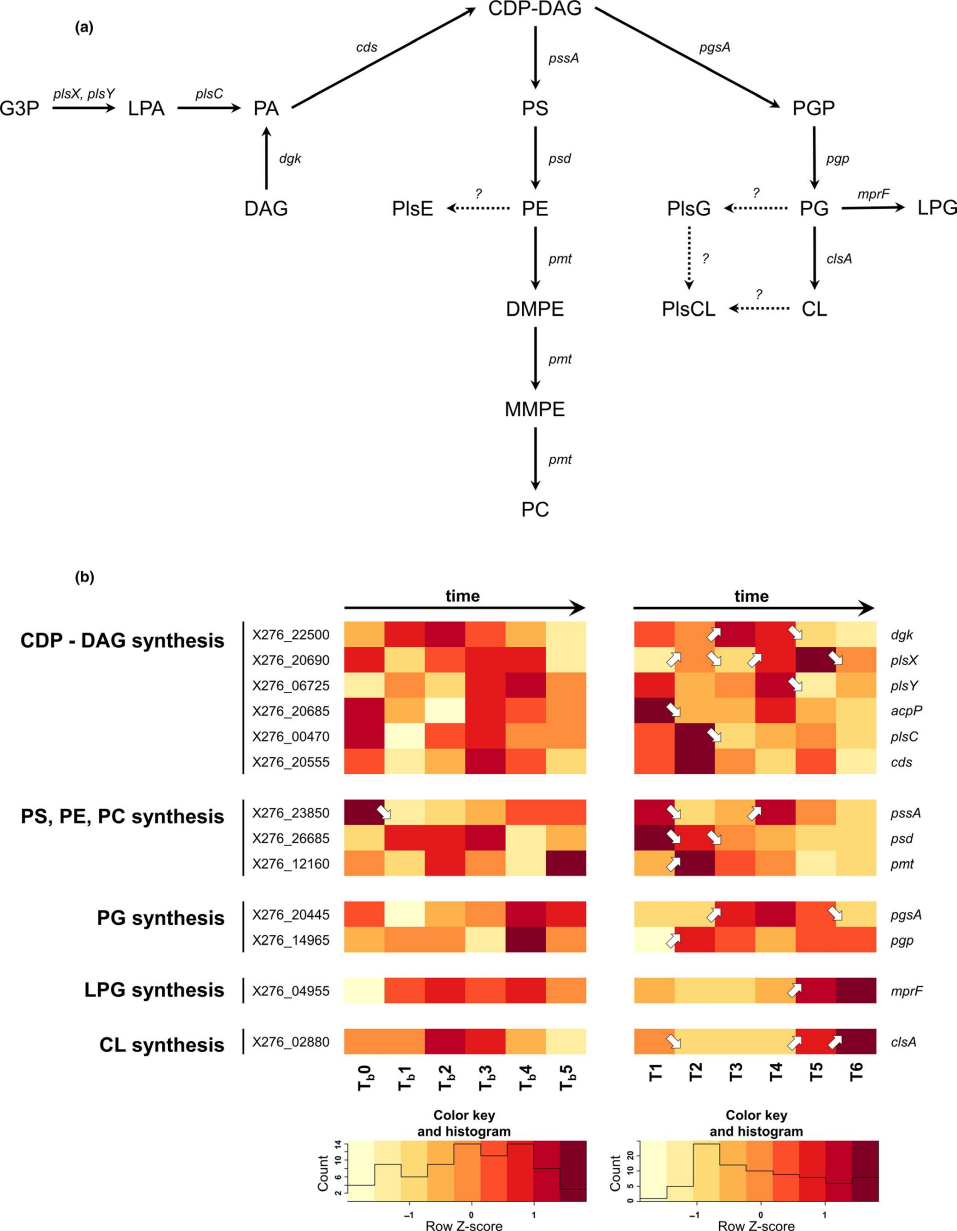
(a) Scheme of the putative membrane phospholipid and plasmenyllipid biosynthetic pathway in *C. beijerinckii* NRRL B‐598. (b) Comparison of expression changes in genes involved in membrane phospholipid synthesis during standard and butanol shocked ABE fermentations (arrows ↗ and ↘ indicate statistically significant (*p*‐adj <0.001, Benjamini–Hochberg correction) upregulation and downregulation of related gene transcription; if there is no arrow in the figure, transcription was not changed significantly). CDP‐DAG, cytidine diphosphate‐diacylglycerol; CL, cardiolipin; DAG, diacylglycerol; DMPE, dimethyl phosphatidylethanolamine; G3P, glycerol‐3‐phosphate; LPA, lysophosphatidic acid; LPG, lysyl‐phosphatidylglycerol; MMPE, monomethyl phosphatidylethanolamine; PA, phosphatidic acid; PC, phosphatidylcholine; PE, phosphatidylethanolamine; PG, phosphatidylglycerol; PGP, phosphatidylglycerol‐phosphate; PlsCL, Plasmenylcardiolipin; PlsE, Plasmenylethanolamine; PlsG, Plasmenylglycerol; PS, phosphatidylserine. For genes description and RPKM values of standard and shocked fermentation, see Table A7

**Figure 7:**
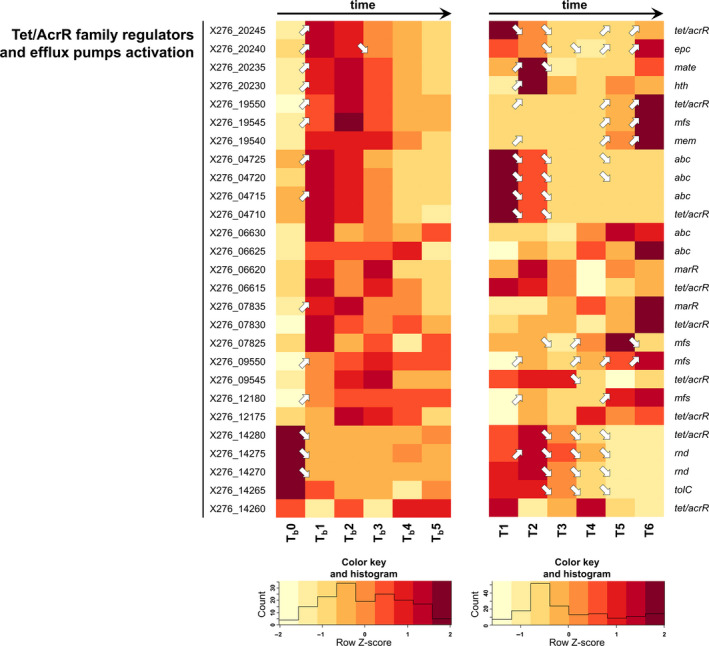
Comparison of expression changes in genes coding for putative efflux pumps and their putative Tet/AcrR regulators during butanol shocked and standard ABE fermentations. (arrows ↗ and ↘ indicate statistically significant (*p*‐adj <0.001, Benjamini–Hochberg correction) upregulation and downregulation of related genes transcription; if there is no arrow in the figure, transcription was not changed significantly). For genes description, putative operon organization, RPKM values of standard and shocked fermentation, and heatmap of standard fermentation see Table A8

Samples for transcriptional analysis were taken at different times in both standard and shocked fermentation because of the different intentions of both experiments. For standard fermentation, sampling times of 3.5 h (fully acidogenic culture, time point T1), 6 h (approx. metabolic switch, T2), 8.5 h (early solventogenic culture, T3), 13 h (mid‐solventogenesis, T4), 18 h (late solventogenic culture, early sporulation, T5), and 23 h (stationary phase of cultivation, sporogenesis, T6) were chosen to cover all main events of biphasic ABE fermentation (the data corresponding to individual samples for RNA‐Seq are given in blue in Table A1). For shocked fermentation, one sample was taken before butanol addition, that is, at 6 h and further early and late responses to butanol shock were determined in samples taken 0.5, 1, 2, 4, and 6 h after the shock (the data corresponding to individual samples for RNA‐Seq are given in red in Table A1). Transcriptional data for selected genes are given in Figures 1, 2, 3, 4, 5, 6, 7 in the form of a heatmap(s) using a Z‐score related to the average expression of each gene and showing results of differential expression analysis (*p*‐adj <0.001, Benjamini–Hochberg correction) in the form of arrows (↗ for upregulation, ↘ for downregulation and without a symbol for a nonsignificant change). Heatmaps for both standard and shocked fermentations are shown for genes whose transcriptional changes were not studied in our previous work. Here, only the heatmap from shocked fermentation is shown. In Tables A2–A8, gene descriptions, average RPKM values (reads per kilobase per million mapped reads) calculated from all replicates are given for both standard and shocked fermentations, including other appropriate information, which can be used for comparison of results obtained in both studies.

The effect of butanol on the population was the most pronounced 30 min and 1 h after the shock when only 25 and 16% of cells in the population displayed vital functions and this was also confirmed by the transcriptional analysis when the biggest changes in genes involved in central metabolism (see Figure 1) were observed immediately after butanol addition. A considerable number of glycolytic genes were downregulated within the first hour, including genes for phosphofructokinase *pfk* (X276_01370) and pyruvate kinase *pyk* (X276_01375) responsible for regulating the flux through glycolysis, suggesting a decreasing rate of glucose utilization. However, it should be noted that during standard ABE fermentation, expression of glycolytic genes also decreased with the transition from acidogenesis to solventogenesis (Patakova et al., 2019; Shi & Blaschek, 2008), probably as a result of a decrease in growth rate as the culture entered the solventogenic phase. Noteworthy, among these genes, phosphoglycerate mutase *gpm* (X276_23710) and ATP‐dependent 6‐phosphofructokinase *pfk* (X276_21855) were upregulated in a reaction to the artificial addition of butanol. The solventogenesis continued after the shock, and 2 h after the shock, the average solvent productivity (0.27 g/L/h) was comparable with the value obtained at a similar time in standard cultivation (see Table A1).

Downregulation of the genes involved in glucose uptake via the phosphotransferase system (PTS) was recorded coincidently with a decrease in the expression of glycolytic genes (Figure 1). In a non‐challenged culture (Patakova et al., 2019), expression of PTS‐related genes was upregulated again 2.5 h after the metabolic switch. In the case of the challenged culture, no such PTS upregulation was detected within an analyzed interval of 6 h. An exception was kinase *hprK* (X276_19670), putatively responsible for regulating PTS‐mediated carbohydrate uptake by the control of phosphorylation of PtsH (a cytoplasmic, histidine‐containing protein involved in phosphate transfer from phosphoenolpyruvate to imported sugar). *HprK* was upregulated after butanol shock and remained highly transcribed. Additionally to the PTS‐sugar transport, glucose can also be taken up by the alternative non‐PTS system in *C. beijerinckii* strains (Lee et al., 2005), where ATP‐dependent glucokinase takes over the role of phosphoenolpyruvate dependent phosphorylation when PTS is repressed (Lee & Blaschek, 2001). This substitutability was not confirmed for butanol‐challenged cells, and the only identified putative glucokinase (X276_01140) was not differentially expressed over the time course of the experiment.

The expression of genes *pta*, *ack*, and *ptb*, *buk* for utilization of acetyl‐CoA and butyryl‐CoA, respectively, to their corresponding acids was decreased after butanol addition, and the metabolism was redirected to solvent production. Both the *sol* (solvent synthesis) operon (X276_06740–X276_06755) and *bcs* (butyryl‐CoA synthesis) operon (X276_25200–X276_25220) were coincidently upregulated in an immediate response to butanol shock, see Figure 1.

Although the culture survived the butanol shock, the influence of butanol addition on the physiological state of the population was still prominent in the population 6 h after the shock. A viability test revealed only 44% of viable cells compared to 68% in standard fermentation. Also, growth was slower, and slower acid utilization was reflected in higher total acid concentration (4.4 g/L versus 3.5 g/L) and lower pH (5.0 versus 5.4), see Table A1. Solvent‐stress related downregulation of glycolysis genes with enhanced expression of butyryl‐CoA synthesis genes has previously been described for *C. acetobutylicum* 824(pGROE1) (Tomas et al., 2004). However, while in our experiment, *ptb* (X276_25645) and *buk* (X276_25640) were repressed, expression in *C. acetobutylicum* followed a similar pattern to butyryl‐CoA pathway genes. In another study, Alsaker et al. (2010) recorded transcriptional changes in *C. acetobutylicum* ATCC 824 glycolytic genes only after acetate and butyrate stress with no statistically relevant responses to butanol, and likewise, experimental butanol addition to chemostat cultured *C. acetobutylicum* cells in acidogenic growth phase did not cause upregulation of any butanol production genes (Janssen et al., 2012).

### 3.1 Sporulation, Agr quorum sensing, and putative polyketide synthesis

After 24 h of fermentation (i.e., 18 h from butanol shock), the population was in a similar state as was the population in standard fermentation at a similar cultivation time, see Table A1. After 35 and 49 h from fermentation start, there was one significant difference between standard and shocked fermentation; while the standard population underwent a complete sporulation cycle and formed mature, free spores, as determined using flow cytometry (6 and 7% of the cells), the shocked population did not form spores and it was not even possible to observe typically swollen (granulose containing) *Clostridium*‐like cells under the microscope, as was shown by Sedlar et al. (2019). This is in complete contradiction with results obtained for *C. acetobutylicum* ATCC 824 where butanol shock did not affect sporulation (Alsaker et al., 2010). In the case of granulose formation, expression of *glgC* and *glgD* genes was slightly decreased after butanol addition, see Figure 2, which means that the conversion of glucose‐1‐P to ADP‐glucose was limited (see putative granulose formation pathway in Patakova et al., 2019). During standard ABE fermentation, coordinated and timely expression of sigma factors SigH, SigF, SigE, SigG, and SigK involved in different sporulation phases (Patakova et al., 2019) were observed, while in the case of butanol shock, all sporulation‐related sigma factors, except for SigK, were downregulated the same as Spo0A, the known master sporulation regulator. Putative SigH repressor, AbrB (X276_0125) was, in contrast, upregulated, which is in complete agreement with the expectation for cultivation with sporulation inhibition.

Sporulation might be regulated by the Agr quorum sensing (QS) system, and three such putative systems were found in *C. beijerinckii* NRRL B‐598 (Patakova et al., 2019). Notably, while two of them were downregulated after butanol addition, the third one, including genes from (X276_23490) to (X276_23505), was upregulated (see Figure 2). Therefore, which sporulation‐related genes might be influenced by quorum sensing was examined. For *C. acetobutylicum*, it was found (Herman et al., 2017) that polyketides can directly control sporulation, but no orthologs of the *C. acetobutylicum* polyketide genes were found in the genome of *C. beijerinckii* NRRL B‐598. Nevertheless, a hybrid polyketide system containing both polyketide synthase (PKS) genes and non‐ribosomal peptide synthase (NRPS) genes was found in the closely related strain, *C. beijerinckii* NCIMB 8052, which included *nrps* genes (cbei_0250) and (cbei_0251) and *fabD* gene (cbei_0257) encoding ACP‐S‐malonyltransferase (Letzel et al., 2013). Two clusters of putative hybrid polyketide biosynthesis‐related genes (A from X276_04685 to X276_04700 and B from X276_07690 to X276_07725) were found in *C. beijerinckii* NRRL B‐598. The cluster A (X276_04685–X276_04700) was transcribed during standard ABE fermentation until the 13th hour of cultivation, see Figure 3, that is, till the time when the population reached approx. sporulation stage II. In contrast, butanol shock caused downregulation of the genes. The other gene cluster B (X276_07690–X276_07725) included *nrps* genes (X276_07725) and (X276_07700); however, the level of transcription of this gene cluster, as assessed using RPKM values, was low during both standard and shocked ABE fermentations, and therefore, it is shown only in Table A4.

Besides, a larger gene cluster C (from X276_10315 to X276_10460) putatively responsible for an unknown polyketide compound(s) was found in the genome of *C. beijerinckii* NRRL B‐598. The genes probably have no orthologs in *C. beijerinckii* NCIMB 14988 and *C. beijerinckii* NCIMB 8052. From the transcription patterns of these genes, it seems that suppression of their transcription might be related to the presence of butanol or its concentration (Figure 3). The cluster might originally have been an insertion sequence (transposon) acquired in a soil environment from other bacteria, similarly as described for other clostridia (Behnken & Hertweck, 2012). Individual genes within the cluster, as well as the whole cluster, require further study but interestingly, an intrinsic transcription regulator LoaP (X276_10350) was found. This regulator, studied in *Bacillus amyloliquefaciens* (Goodson et al., 2017), is often found in Firmicutes, including clostridia, and regulates the biosynthesis of secondary metabolites, especially polyketides.

### 3.2 Stress response

In the overall comparison, while during standard ABE fermentation, a maximum butanol concentration of 7.1 g/L was reached, the shocked population produced approximately 4.0 g/L butanol but the total butanol concentration (including added butanol) was 8.2 g/L.

### 3.3 Heat shock proteins

Production of heat shock proteins (HSPs) represents one of the mechanisms responsible for adaption to butanol shock, as was shown several times previously in clostridia (Liao et al., 2017; Mann et al., 2012; Patakova et al., 2019; Tomas et al., 2003). It was found that HSPs are one of the significantly upregulated groups of genes in *C. beijerinckii* B‐598 during butanol shock, as was shown using a gene ontology enrichment approach (Sedlar et al., 2019). In the global view, expression of different HSPs or proteins connected with reparative functions vary between standard cultivation and butanol challenge in *C. beijerinckii* B‐598, see Figure 5.

The expression of the main class I HSPs (Patakova et al., 2019) negative regulator gene *hrc*A (X276_22580) was activated directly after butanol shock, with genes encoding other HSPs of this group, such as DnaKJ, GrpE, or GroESL. This was in variance with the previous assumption that class I HSPs were negatively regulated by HrcA. On the contrary, expression of the gene encoding alternative sigma factor SigI (X276_17720) decreased noticeably after butanol addition, indicating that SigI is probably not able to substitute for the function of class II HSPs in clostridia, as was postulated previously (Kim et al., 2007; Patakova et al., 2019; Zuber et al., 2001).

The class III HSPs negative regulator *cts*R (X276_26065) was also activated after butanol shock, with other genes encoding proteins included in the class III group, such as *clp*X (X276_19855) and *clp*P (X276_19860). This is also in variance with previous findings in *C. beijerinckii* NRRL B‐598 (Patakova et al., 2019). Except for those described, many uncategorized HSPs and other reparative proteins could be found in the *C. beijerinckii* B‐598 genome, such as HptG (X276_05050), Asp23 (X276_18540), Tig (X276_19865), or RadA (X276_26035); expression of genes encoding these proteins also varied as can be seen in Figure 5.

### 3.4 Cell membrane changes

Disturbance of cytoplasmic membranes is a serious consequence of alcohol action on the cell. Butanol and other solvents cause membrane destabilization by increasing its fluidity and generally causing membrane protein damage. In response to this condition, bacterial cells can modify the ratio of saturated/unsaturated or *cis*/*trans* fatty acids (FAs) in their membrane to prevent destabilization (Ingram & Buttke, 1985; Lepage et al., 1987; Sardessai & Bhosle, 2002; Sikkema et al., 1995). Because clostridia generally lack the enzyme *cis*/*trans* isomerase, the mechanism of *cis*/*trans* FAs shift cannot be included in the butanol stress response. On the other hand, the ratio of saturated/unsaturated FAs can play a significant role (Kolek et al., 2015; Lepage et al., 1987; Vollherbst Schneck et al., 1984). As was described previously, the next group of FAs that may help with membrane stabilization in clostridia and other bacteria are cyclopropanated FAs (Chang & Cronan, 1999; Kolek et al., 2015; Zhao et al., 2003). Over‐expression of the gene for cyclopropane fatty acid synthase (*cfa*) in *C. acetobutylicum* ATCC 824 has been demonstrated as one potential way to directly increase butanol resistance (Zhao et al., 2003).

The *C. beijerinckii* NRRL B‐598 fatty acid biosynthetic cluster is organized in *fab* operon(s), a format typical for *C. beijerinckii* species, as well as a *Bacillus* model (De Mendoza et al., 2002; Patakova et al., 2019), which differ slightly from the model strain *C. acetobutylicum* ATCC 824 (Tomas et al., 2004). During standard cultivation in the same arrangement, the highest expression of genes from the fatty acid biosynthetic cluster was detected at 3.5 h, with subsequent downregulation and the next activation at times 8.5 and 13 h (Patakova et al., 2019). In the case of butanol challenge, sudden downregulation of fatty acid biosynthetic genes was observed directly after butanol addition, see Figure 5. Restored expression was evident after the next four hours, which correlates well with the restoration of cellular growth. In contrast to genes of the *fab* operon(s), *cfa* (X276_00620) was upregulated strongly after butanol addition. This strongly supports the hypothesis that the production of cyclopropanated fatty acids represents an essential mechanism for protecting the cell membrane and restoring its function during stress caused by solvents (Chang & Cronan, 1999; Patakova et al., 2019; Pini et al., 2009; Zhao et al., 2003).

The proportional representation of each fraction of phospholipids most probably also has a fundamental influence on membrane stability and function during butanol shock. Unfortunately, detailed lipidomic studies are rarer compared to genomic and proteomic analyses in clostridia, and studies describing any interconnection between lipid synthesis and gene expression are missing completely. The assumed biosynthetic pathway for the main classes of membrane phospholipids is shown in Figure 7a. Unfortunately, several steps in their biosynthesis are still only hypothetical or even completely unknown, especially anoxygenic synthesis of plasmalogen lipids (see below).

Genes involved in membrane phospholipid precursors such as DAG, PA, and CDP‐DAG were expressed with local maxima at times T1 (early acidogenesis) and T4 (mid‐solventogenesis) during standard cultivation conditions, see Figure 7b. The gene encoding phosphatidylserine synthase (X276_23850) also had a similar expression pattern at times T1 and T4, reflecting a similar situation in terms of pH as well as butyrate titer, and possibly ideal conditions for growth. Stronger expression of genes encoding Psd and Pmt, involved in PE and PC synthesis, is retarded compared to previous genes, which agrees with the assumption that their expression is regulated by the concentration of the reaction substrate. PG biosynthetic genes are expressed maximally at times T3–T5, followed by genes involved in cardiolipin (di‐phosphatidylglycerol) and LPG synthesis.

Butanol shock led to a decrease in expression of several genes involved in the synthesis of different phospholipid classes/precursors, including LPA, PA, PS, or PC. On the other hand, *dgk*, *psd*, *pgp*, and *mprF* had stronger expression directly after butanol addition. Higher expression of genes involved in CL and LPG synthesis seemed to correspond with higher butanol titers in both experiments, which is in accordance with a previous lipidomic study conducted on *C. beijerinckii* NRRL B‐598 (Kolek et al., 2015) as well as older studies conducted with *C. butyricum* (MacDonald & Goldfine, 1991).

The occurrence of a large fraction of plasmalogens from lipids in the membrane is a very specific attribute of clostridia and some other anaerobic bacteria because plasmalogens are completely missing in aerobic and facultative aerobic bacteria. Plasmalogens are diacyl phospholipids containing alk‐1'‐enyl ether‐linked hydrocarbon chains in position sn‐1 of phospholipids as well as glycerol glycolipids. This unusual family of lipids probably also contributes to the overcoming of butanol/solvent stress, as was described in several pilot studies (Goldfine, 2010; Goldfine & Johnston, 2005; Han & Gross, 1990; Johnston et al., 1987), including one study using strain *C. beijerinckii* NRRL B‐598 as a model (Kolek et al., 2015). The plasmalogen biosynthetic pathway differs fundamentally in anaerobic bacteria compared with other organisms that can synthesize plasmalogens by an oxygen‐dependent pathway (Goldfine, 2010). The substrate for plasmalogen synthesis in clostridia is most likely diacyl forms of lipids, as was demonstrated previously using radioactive labeling (Baumann et al., 1965; Koga & Goldfine, 1984); however, the direct pathway and its genetic background are still unknown.

### 3.5 Efflux

Active butanol efflux seems to be another option for cells to survive and maintain all necessary functions after butanol shock; the expression of putative butanol exporter genes was therefore also studied. At first, attention was focused on putative efflux genes that might be regulated by the TetR/AcrR family of transcriptional regulators because these regulators had been described in the group of genes, the expression of which, was upregulated by butanol shock (Sedlar et al., 2019). This seems to be counter‐intuitive because typically the TetR/AcrR family of transcriptional regulators is known as one‐component transcriptional repressors (Deng et al., 2013) although there have already been cases described in which these regulators acted as activators (Murarka et al., 2019; Nguyen Le Minh et al., 2015). Seven gene clusters were found that might be involved in butanol efflux, see Figure 7, (for operon analysis, see Table A8), in which the respective genes appeared to be activated by the TetR/AcrR family of transcriptional regulators. Interestingly, transporters belonging to different families (MFS, MATE, and ABC transporters) were activated after butanol shock, and therefore, it seems that butanol efflux might be nonspecific. Contrary to expectations, other genes encoding the putative RND family transporter (from X276_14265 to X276_14275) were found, that are possibly also regulated by TetR/AcrR family regulators, for which transcription was downregulated by butanol addition. It was assumed that RND efflux pumps were the best candidates for solvent efflux and an AcrB pump engineered in *E. coli* could even transport butanol, which does not correlate with our findings (Mukhopadhyay, 2015) (Fisher et al., 2014).

## 4 CONCLUSIONS

Comparative analysis of transcriptomes obtained during standard and shocked ABE fermentations focused on transcriptional changes of individual genes elicited by butanol addition to the growing population in the acidogenic phase resulted in surprising findings. While glucose uptake, glycolytic, and butyryl‐CoA synthesis genes were downregulated immediately after butanol shock, *sol* operon genes were upregulated. Granulose formation and sporulation initiation genes, as well as all sporulation‐related sigma factors, were suppressed. Surprisingly, one of three identified putative Agr QS gene clusters was upregulated while the remaining two were downregulated after butanol shock. From two‐hybrid polyketide gene clusters, one was actively transcribed during both standard and shocked fermentation, and was downregulated after butanol addition. Although upregulation of HSPs genes after butanol shock was expected, their transcription pattern varied from that obtained during standard fermentation. Stabilization of cell membranes in the presence of butanol is probably mediated by cyclopropanation of fatty acids and biosynthesis of cardiolipins and plasmalogen forms of these phospholipids. Some genes encoding putative efflux pumps, which might be regulated by Tet/AcrR transcriptional regulators after butanol addition, were identified.

The take‐home message from the comparative analysis is that future success in increasing butanol tolerance and production by clostridia requires a deeper understanding of the regulation of both individual genes/gene clusters and population stress responses. From the global view, the manipulation of selected regulators or influencing signaling molecules offers the achievement of the goal more efficiently and faster compared to the manipulation of individual genes. However, insufficient knowledge prevents the application of this approach. The article outlines the questions that we should focus on to move forward such as What is the functioning of positive feedback when butanol addition elicits upregulation of *sol* operon genes? Can the key factor in population regulation be population density mediated through the quorum sensing phenomenon? Is it possible that the population will respond to the same stress differently if the population density is different? What are the key signaling molecules involved in the process? Polyketides? Also, progress in the field of butanol production by clostridia is inevitably associated with fundamental research. There are still white spots waiting for their discoverers, such as What is the anaerobic way of plasmalogen synthesis and how is it regulated? Can butanol efflux alleviate butanol stress? Is there anything as specific as butanol efflux? Clarification of these questions will benefit from further advanced transcriptome studies.

## CONFLICTS OF INTEREST

None declared.

## AUTHOR CONTRIBUTIONS

Petra Patáková involved in conceptualization, funding acquisition, and writing–original draft. Jan Kolek involved in investigation and writing–original draft. Katerina Jureckova involved in methodology, visualization, and writing–review and editing. Barbora Branska involved in data curation, investigation and writing–original draft. Karel Sedlar involved in data curation, methodology, and writing–original draft. Maryna Vasylkivska involved in investigation, methodology, and writing–original draft. Ivo Provaznik involved in funding acquisition, supervision, and writing–review and editing.

## ETHICS STATEMENT

None required.

## Supporting information

Appendix S1‐S8

## Data Availability

The genome assembly referred to in this paper is version CP011966.3, available from the NCBI GenBank database: https://www.ncbi.nlm.nih.gov/nuccore/CP011966.3. The RNA‐Seq sequencing data sets of separate replicates collected during standard and shocked fermentation are available in the NCBI Sequence Read Archive (SRA) under accession number SRP033480: https://trace.ncbi.nlm.nih.gov/Traces/sra/sra.cgi?study=SRP033480.
