# Supplementary material for: Deeper below the surface—transcriptional changes in selected genes of Clostridium beijerinckii in response to butanol shock
Source: Microbiologyopen. 2020 Dec 14;10(1):e1146. doi: 10.1002/mbo3.1146 (PMC7884928; doi:10.1002/mbo3.1146)
Supplement: Supplementary file 1 — Appendix S1‐S8 [file MBO3-10-e1146-s001.docx]

| APPENDIX 1  **Table A1 Standard versus shocked ABE fermentation, comparison of fermentation parameters** |  |
| --- | --- |

| Sample name | Fermen-tation  type | Cultivation  Time (h) | Time after butanol shock (h) | pH | OD_600_ | Butanol (g/l) | Total  Solvents (TS) (g/l) | Butyric acid (g/l) | Total acids  (g/l) | Cytometry analysis  viable/non-viable/  spores  (%) | Glucose consumption  rate- r_glc_ (g/l/h) | Solvents  Productivity – p_solv_  (g/l/h) |
| --- | --- | --- | --- | --- | --- | --- | --- | --- | --- | --- | --- | --- |
| **T1** | **Standard** | **3.5** | **-** | **5.4 ± 0.1** | **0.9 ± 0.1** | **0** | **0** | **0.6 ± 0.1** | **3.6 ± 0.2** | **55/45/0** | **0.37 ± 0.06** | **0** |
|  |  |  |  |  |  |  |  |  |  |  |  |  |
| **T2** | **Standard** | **6** | **-** | **5.0 ± 0.0** | **1.6 ± 0.1** | **0.3 ± 0.0** | **0.3 ± 0.0** | **1.1 ± 0.1** | **4.3 ± 0.2** | **73/27/0** | **0.42 ± 0.05** | **0.05 ± 0.00** |
| ***Tb0*** | ***Shocked***  ***Prior shock*** | ***6*** | ***-*** | ***5.0 ± 0.2*** | ***1.5 ± 0.1*** | ***0.2 ± 0.0*** | ***0.2 ± 0.0*** | ***1.2 ± 0.1*** | ***4.5 ± 0.2*** | ***76/24/0*** | ***0.37 ± 0.04*** | ***0.03 ± 0.00*** |
|  |  |  |  |  |  |  |  |  |  |  |  |  |
| ***Tb1*** | ***Shocked***  ***After shock*** | ***6.5*** | ***0.5*** | ***5.0 ± 0.2*** | ***1.5 ± 0.1*** | ***4.4 ± 0.2***  ***(0.2)^*^*** | ***4.4 ± 0.2*** | ***1.2 ± 0.1*** | ***4.5 ± 0.2*** | ***25/75/0*** | ***-*** | ***-*** |
| ***Tb2*** | ***Shocked*** | ***7*** | ***1*** | ***5.0 ± 0.2*** | ***1.6 ± 0.2*** | ***4.5 ± 0.2***  ***(0.3)^*^*** | ***4.6 ± 0.2*** | ***1.2 ± 0.1*** | ***4.5 ± 0.2*** | ***16/84/0*** | ***0.68 ± 0.05*** | ***0.27 ± 0.03*** |
|  |  |  |  |  |  |  |  |  |  |  |  |  |
| **T3** | **standard** | **8.5** | **-** | **5.0 ± 0.2** | **2.3 ± 0.2** | **0.8 ± 0.2** | **1.2 ± 0.3** | **1.2 ± 0.1** | **4.5 ± 0.2** | **60/40/0** | **0.80 ± 0.08** | **0.28 ± 0.03** |
| ***Tb3*** | ***Shocked*** | ***8*** | ***2*** | ***5.0 ± 0.2*** | ***1.8 ± 0.2*** | ***4.5 ± 0.2***  ***(0.3)^*^*** | ***4.6 ± 0.2*** | ***1.2 ± 0.1*** | ***4.5 ± 0.2*** | ***40/60/0*** | ***0.52 ± 0.05*** | ***0.27 ± 0.04*** |
|  |  |  |  |  |  |  |  |  |  |  |  |  |
| ***Tb4*** | ***Shocked*** | ***10*** | ***4*** | ***5.0* ± 0.2** | ***2.1* ± 0.3** | ***4.7* ± 0.2**  ***(0.5)^*^*** | ***4.9* ± 0.3** | ***1.2* ± 0.1** | ***4.5* ± 0.2** | ***32/68/0*** | ***0.56* ± 0.06** | ***0.35* ± 0.03** |
|  |  |  |  |  |  |  |  |  |  |  |  |  |
| **T4** | **Standard** | **13** | **-** | **5.4 ± 0.2** | **4.0 ± 0.3** | **2.3 ± 0.2** | **3.8 ± 0.3** | **0.7 ± 0.1** | **3.5 ± 0.2** | **68/32/0** | **0.84 ± 0.12** | **0.54 ± 0.04** |
| ***Tb5*** | ***Shocked*** | ***12*** | ***6*** | ***5.0 ± 0.2*** | ***2.6 ± 0.2*** | ***4.9 ± 0.2 (0.7)^*^*** | ***5.2 ± 0.3*** | ***1.2 ± 0.1*** | ***4.4 ± 0.3*** | ***44/56/0*** | ***0.61 ± 0.11*** | ***0.50 ± 0.01*** |
|  |  |  |  |  |  |  |  |  |  |  |  |  |
| **T5** | **Standard** | **18** | **-** | **5.3 ± 0.2** | **8.4 ± 0.3** | **4.1 ± 0.2** | **6.5 ± 0.3** | **0.7 ± 0.1** | **3.5 ± 0.2** | **60/40/0** | **1.70 ± 0.22** | **0.51 ± 0.04** |
|  |  |  |  |  |  |  |  |  |  |  |  |  |
| **T6** | **Standard** | **23** | **-** | **5.3 ± 0.2** | **9.7 ± 0.6** | **5.4 ± 0.2** | **8.2 ± 0.3** | **0.9 ± 0.1** | **3.3 ± 0.2** | **50/49/1** | **1.52 ± 0.21** | **0.44 ± 0.06** |
| ***Tb24*** | ***Shocked*** | ***24*** | ***18*** | ***5.4 ± 0.2*** | ***4.4 ± 0.3*** | ***7.5 ± 0.1***  ***(3.3)^*^*** | ***9.1 ± 0.2*** | ***0.6 ± 0.1*** | ***4.1 ± 0.2*** | ***44/56/0*** | ***0.63 ± 12*** | ***0.24 ± 0.04*** |
| **T33** | **Standard** | **33** | **-** | **5.2 ± 0.2** | **9.7 ± 0.6** | **7.1 ± 0.2** | **10.7 ± 0.3** | **0.9 ± 0.1** | **3.4 ± 0.2** | **11/83/6** | **0.53 ± 0.09** | **0.25 ± 0.03** |
| ***Tb29*** | ***Shocked*** | ***35*** | ***29*** | ***5.6 ± 0.2*** | ***6.2 ± 0.5*** | ***8.2 ± 0.1 (4.0)^*^*** | ***11.0 ± 0.2*** | ***1.0 ± 0.1*** | ***3.9 ± 0.2*** | ***10/90/0*** | ***0.21 ± 0.04*** | ***0.17 ± 0.02*** |
|  |  |  |  |  |  |  |  |  |  |  |  |  |
| **T48** | **Standard** | **48** | **-** | **5.2 ± 0.1** | **7.9 ± 0.6** | **7.1 ± 0.2** | **10.7 ± 0.3** | **1.0 ± 0.2** | **3.4 ± 0.3** | **0/93/7** | **-** | **-** |
| ***Tb49*** | ***Shocked*** | ***49*** | ***43*** | ***5.6 ± 0.1*** | ***6.2 ± 0.5*** | ***8.2 ± 0.3***  ***(4.0)^*^*** | ***11.1 ± 0.4*** | ***1.0 ± 0.2*** | ***3.9 ± 0.3*** | ***0/100/0*** | ***-*** | ***-*** |

Yellow highlighted data display results of analyses during shocked ABE fermentation. Butanol shock was caused by the addition of 0.5 % v/v of butanol to *C. beijerinckii* NRRL B-598 at the 6^th^ hour of batch fermentation after sampling (i.e. data for Tb0 display the status of the fermentation before the shock, while the first sample after the shock was taken after 30 min and the corresponding results are shown for Tb1).

Data given in blue/red correspond with the samples used for RNA-Seq analysis during standard/shocked ABE fermentation. Samples are labeled T1-T6 and Tb0-Tb5 for standard and shocked fermentation, respectively. The remaining data in black describe the further progression of both fermentations but samples taken at the respective times were not used for RNA-Seq analysis.

For a better reader`s orientation, data obtained from samples taken at similar fermentation times during standard and shocked cultivations are framed in red.

In both cases (standard and shocked cultivation), TYA medium, see Materials and Methods, was used.

**Butanol:** ^*^The items in brackets represent the concentration of butanol formed i.e. from the determined total butanol concentration was subtracted the added butanol concentration i.e. 4.2 g/l in all cases;

**Total solvents** (TS) means the sum of concentrations of butanol, acetone, and ethanol irrespective of whether the solvent was formed by cells or added to the bioreactor.

**Total acids** means the sum of concentrations of acetate, butyrate, and lactate irrespective of whether the compound was formed by cells or added to the bioreactor (ammonium acetate in the concentration of 3 g/l was part of the TYA fermentation medium).

**Average glucose consumption rate (g/l/h)** was calculated as r_glc_= $\frac{X_{{glc}_{i}}-X_{{glc}_{i+1}}}{t_{i+1}-t_{i}}$ where $X_{{glc}_{i}}$and $X_{{glc}_{i+1}}$are concentrations of glucose (in g/l) determined in the samples taken at times t_i_; t_i+1_ respectively. As an example, r_glc_ for T1 (see Table 1) was calculated using the glucose concentration determined after inoculation of the culture medium in the bioreactor ($X_{{glc}_{i}}$) and the glucose concentration in the sample was taken at 3^rd^ h of the cultivation $X_{{glc}_{i+1}}$ while the difference was divided by the time difference $t_{i+1}-t_{i}$ in hours.

**Average solvents productivity (g/l/h)** was calculated as p_solv_ = $\frac{{TS}_{i+1}-{TS}_{i}}{t_{i+1}-t_{i}}$ where ${TS}_{i+1}$ and ${TS}_{i}$ are sums of the butanol, acetone, and ethanol concentrations (in g/l) determined at times t_i_; t_i+1._ In the case of shocked fermentation, the butanol concentration of 4.2 g/l, corresponding to the amount of butanol added at butanol shock, was subtracted from all TS values.

For **Cytometry analysis** see Materials and Methods.

| **Table A2: Central metabolism - RPKM values** | | | | | |  |  |  |  |  |  |  |  |  |  |
| --- | --- | --- | --- | --- | --- | --- | --- | --- | --- | --- | --- | --- | --- | --- | --- |
|  |  |  |  | Shocked ABE fermentation | | | | | | Standard ABE fermentation | | | | | |
|  | Gene | Putative physiological role | Locus tag | T_b_0 | T_b_1 | T_b_2 | T_b_3 | T_b_4 | T_b_5 | T1 | T2 | T3 | T4 | T5 | T6 |
| **Glucose uptake via PTS** | | |  |  |  |  |  |  |  |  |  |  |  |  |  |
|  | *ptsI* | PTS enzyme I | X276_25680 | 825.52 | 401.47 | 472.06 | 502.16 | 515.17 | 531.33 | 776.87 | 705.95 | 688.85 | 593.11 | 480.70 | 390.42 |
|  | *ptsH* | histidine-containing protein HPr | X276_20425 | 2452.31 | 2280.43 | 1924.47 | 2148.61 | 2404.04 | 2630.23 | 3987.94 | 3452.56 | 4641.42 | 4659.73 | 2215.03 | 2119.18 |
|  | *hprK* | HPr kinase/phosphorylase | X276_19670 | 370.87 | 428.66 | 412.70 | 502.00 | 510.37 | 486.96 | 307.60 | 416.09 | 364.90 | 215.31 | 174.30 | 199.31 |
|  | *manIIAB* | PTS enzyme EII AB subunit | X276_23245 | 1173.92 | 968.38 | 812.73 | 788.67 | 782.91 | 835.94 | 1785.32 | 1338.48 | 2908.85 | 2206.96 | 1923.33 | 1013.81 |
|  | *manIIC* | PTS enzyme EII C subunit | X276_23240 | 935.91 | 668.98 | 522.05 | 479.65 | 489.94 | 541.18 | 1735.19 | 1150.63 | 2541.38 | 1671.25 | 1509.51 | 836.48 |
|  | *manIID* | PTS enzyme EII D subunit | X276_23235 | 1165.42 | 792.68 | 592.34 | 583.54 | 627.45 | 659.04 | 2537.29 | 1492.69 | 3345.80 | 2169.81 | 2042.00 | 1069.40 |
|  | *manIID* | PTS enzyme EII D subunit | X276_02990 | 1521.12 | 898.59 | 707.43 | 586.80 | 533.24 | 422.66 | 1905.12 | 1724.56 | 2054.24 | 1694.68 | 840.97 | 682.41 |
|  | *manIIC* | PTS enzyme EII C subunit | X276_02985 | 1821.99 | 1087.51 | 805.73 | 682.84 | 604.36 | 484.42 | 2251.28 | 2108.78 | 2369.65 | 1743.91 | 967.33 | 840.49 |
|  | *manIIB* | PTS enzyme EII B subunit | X276_02980 | 2010.61 | 1175.08 | 798.42 | 603.54 | 597.25 | 469.85 | 2363.98 | 2446.86 | 2714.72 | 1877.13 | 948.08 | 794.11 |
|  | *manIIAB* | PTS enzyme EII A subunit | X276_02975 | 1974.43 | 1258.41 | 922.63 | 767.36 | 665.01 | 573.25 | 2701.88 | 2460.53 | 2795.10 | 1940.71 | 1085.93 | 926.04 |
| **Glucose uptake via non-PTS** | | |  |  |  |  |  |  |  |  |  |  |  |  |  |
|  | *glcK* | glucokinase | X276_01140 | 166.85 | 140.24 | 145.68 | 164.24 | 146.74 | 156.59 | 174.26 | 214.78 | 236.08 | 295.87 | 269.73 | 174.95 |
| **Glycolysis** | |  |  |  |  |  |  |  |  |  |  |  |  |  |  |
|  | *pgi* | glucose-6-phosphate isomerase | X276_25070 | 1382.87 | 1035.54 | 953.86 | 1138.72 | 1302.56 | 1410.48 | 1343.79 | 1496.22 | 1462.65 | 925.76 | 745.90 | 655.77 |
|  | *pfk* | 6-phosphofructokinase | X276_01370 | 629.13 | 286.90 | 286.08 | 404.21 | 527.88 | 612.14 | 494.51 | 710.71 | 510.32 | 315.77 | 352.46 | 258.27 |
|  | *pfk* | ATP-dependent 6-phosphofructokinase | X276_21855 | 759.75 | 1192.36 | 1143.80 | 1108.67 | 1052.58 | 1102.21 | 733.28 | 938.38 | 961.51 | 1125.13 | 925.23 | 855.34 |
|  | *fba* | fructose-1,6-bisphosphate aldolase, class II | X276_17335 | 6902.91 | 5797.85 | 5076.87 | 5224.97 | 4935.94 | 4823.87 | 4858.31 | 7415.03 | 5547.88 | 2771.26 | 2020.13 | 1898.36 |
|  | *tpi* | triose-phosphate isomerase | X276_23715 | 1685.81 | 1314.12 | 853.14 | 901.09 | 1111.55 | 1302.93 | 1518.60 | 1743.35 | 1013.35 | 375.47 | 730.08 | 519.96 |
|  | *gap* | type I glyceraldehyde-3-phosphate dehydrogenase | X276_23725 | 7923.11 | 5993.32 | 4136.44 | 4565.98 | 5395.91 | 5773.50 | 6139.75 | 8855.02 | 5234.10 | 1648.10 | 3020.89 | 2130.35 |
|  | *pgk* | phosphoglycerate kinase | X276_23720 | 1854.68 | 1443.20 | 1002.42 | 1099.04 | 1338.44 | 1484.17 | 1458.16 | 1880.05 | 1082.30 | 401.77 | 878.95 | 625.90 |
|  | *gpm* | 2,3-bisphosphoglycerate-independent phosphoglycerate mutase | X276_23710 | 666.68 | 889.88 | 793.54 | 805.22 | 654.25 | 636.47 | 809.76 | 740.67 | 511.32 | 259.40 | 302.94 | 307.02 |
|  | *eno* | enolase | X276_23700 | 1986.96 | 1392.68 | 1699.59 | 1791.69 | 1561.63 | 1201.72 | 2558.09 | 2076.35 | 2150.63 | 1675.86 | 1389.76 | 931.59 |
|  | *pyk* | pyruvate kinase | X276_01375 | 1081.25 | 498.79 | 450.73 | 613.88 | 761.66 | 901.62 | 1145.63 | 1132.27 | 685.58 | 435.14 | 509.91 | 317.43 |
|  | *pfo* | pyruvate:ferredoxin (flavodoxin) oxidoreductase | X276_03990 | 8033.75 | 6609.59 | 5811.10 | 5799.71 | 6343.73 | 6454.97 | 6404.99 | 7542.80 | 5125.18 | 2574.39 | 2137.99 | 2107.54 |
|  | *ldh* | L-lactate dehydrogenase | X276_21775 | 320.52 | 122.11 | 181.90 | 222.15 | 256.67 | 230.21 | 426.60 | 350.50 | 357.12 | 323.90 | 243.00 | 222.55 |
|  | *ldh* | L-lactate dehydrogenase | X276_05485 | 145.49 | 168.47 | 190.99 | 218.96 | 217.98 | 198.64 | 130.06 | 183.08 | 226.31 | 171.82 | 192.16 | 174.94 |
| **Acidogenesis** | | |  |  |  |  |  |  |  |  |  |  |  |  |  |
|  | *pta* | phosphate acetyltransferase | X276_20710 | 2203.01 | 1593.12 | 1696.19 | 1794.45 | 1746.42 | 1502.57 | 1479.71 | 2224.38 | 1725.90 | 526.85 | 568.99 | 657.34 |
|  | *ack* | acetate kinase | X276_20705 | 2452.61 | 1576.54 | 1695.49 | 1791.05 | 1724.45 | 1616.41 | 1766.46 | 2584.07 | 2204.54 | 655.09 | 634.01 | 710.71 |
|  | *ptb* | phosphate butyryltransferase | X276_25645 | 1154.36 | 595.41 | 521.75 | 598.22 | 790.10 | 861.79 | 855.91 | 1164.85 | 1333.54 | 698.10 | 460.48 | 355.72 |
|  | *buk* | butyrate kinase | X276_25640 | 1481.10 | 811.42 | 668.01 | 709.85 | 944.33 | 1111.03 | 1360.65 | 1383.43 | 1422.84 | 483.20 | 378.74 | 401.48 |
| **Butyryl-CoA synthesis** | | |  |  |  |  |  |  |  |  |  |  |  |  |  |
|  | *thl* | acetyl-CoA C-acetyltransferase | X276_24605 | 3099.35 | 2478.43 | 1811.49 | 1792.32 | 2250.33 | 2600.29 | 3761.57 | 3043.95 | 3111.73 | 3760.55 | 2154.89 | 1672.52 |
|  | *hbd* | 3-hydroxybutyryl-CoA dehydrogenase | X276_25200 | 2335.48 | 3265.69 | 2274.12 | 1811.81 | 2157.33 | 2811.47 | 2575.67 | 2487.44 | 2508.91 | 2781.77 | 2101.29 | 1775.43 |
|  | *crt* | crotonase | X276_25220 | 1562.12 | 2480.37 | 2174.92 | 1886.27 | 2093.01 | 2316.50 | 1381.98 | 1789.20 | 1994.61 | 1583.12 | 1277.47 | 1293.11 |
|  | *etfA* | electron transfer flavoprotein subunit alpha/FixB family protein | X276_25205 | 2291.41 | 3499.84 | 2735.35 | 2281.57 | 2644.57 | 3064.25 | 2365.88 | 2282.40 | 2347.54 | 2904.16 | 2146.98 | 1948.88 |
|  | *etfB* | electron transfer flavoprotein subunit beta/FixA family protein | X276_25210 | 2627.53 | 3980.29 | 3069.98 | 2623.18 | 3015.51 | 3543.06 | 2681.55 | 2740.51 | 2808.85 | 3158.15 | 2489.34 | 2242.23 |
|  | *bcd* | acyl-CoA dehydrogenase (butyrylCoA dehydrogenase) | X276_25215 | 1851.29 | 2787.25 | 2246.43 | 1950.09 | 2277.35 | 2584.46 | 1714.01 | 1842.04 | 1837.14 | 2023.10 | 1597.06 | 1488.14 |
| **Solventogenesis** | | |  |  |  |  |  |  |  |  |  |  |  |  |  |
|  | *ctfA* | acetyl-CoA--acetoacetyl-CoA transferase subunit alpha | X276_06750 | 9568.03 | 13980.41 | 14195.30 | 11159.50 | 10758.37 | 10348.98 | 2717.94 | 8515.71 | 4774.48 | 4131.62 | 4929.75 | 5151.22 |
|  | *ctfB* | CoA transferase subunit B | X276_06745 | 10109.80 | 14937.12 | 15729.63 | 12701.03 | 12565.43 | 11627.40 | 2930.40 | 9327.01 | 5557.64 | 4589.62 | 5707.56 | 6087.58 |
|  | *adc* | acetoacetate decarboxylase | X276_06740 | 10613.47 | 14923.05 | 14708.37 | 10677.15 | 10336.41 | 9934.53 | 3481.40 | 11009.53 | 6684.13 | 5667.57 | 6788.33 | 7084.50 |
|  | *ald* | aldehyde dehydrogenase EutE; Provisional | X276_06755 | 9034.79 | 13044.46 | 12724.57 | 9297.80 | 8820.54 | 9186.76 | 2695.35 | 9316.34 | 5197.09 | 4511.12 | 4669.95 | 4980.65 |
|  | *bdh* | iron-containing alcohol dehydrogenase | X276_18435 | 1968.19 | 2166.04 | 1724.92 | 1680.31 | 1517.32 | 1652.63 | 1161.99 | 2111.46 | 2038.05 | 1289.64 | 526.61 | 516.95 |
|  | *bdh* | iron-containing alcohol dehydrogenase | X276_15910 | 809.23 | 948.67 | 1010.41 | 1241.65 | 1370.69 | 1625.28 | 154.81 | 793.75 | 1488.30 | 251.60 | 312.40 | 335.38 |
|  | *adh* | bifunctional acetaldehyde-CoA/alcohol dehydrogenase | X276_25300 | 4.91 | 4.15 | 5.34 | 4.88 | 5.07 | 5.80 | 13.92 | 4.18 | 5.61 | 38.90 | 79.40 | 25.82 |
|  |  |  |  |  |  |  |  |  |  |  |  |  |  |  |  |
|  |  |  |  |  |  |  |  |  |  |  |  |  |  |  |  |
| **Table A2 additional information: Central metabolism - Heatmap (standard ABE fermentation)** | | | | | | | | | | | | | | | |
|  |  |  |  | Standard ABE fermentation | | | | | |  |  |  |  |  |  |
|  | Gene | Putative physiological role | Locus tag | T1 | T2 | T3 | T4 | T5 | T6 |  |  |  |  |  |  |
| **Glucose uptake via PTS** | | |  |  |  |  |  |  |  |  |  |  |  |  |  |
|  | *ptsI* | PTS enzyme I | X276_25680 | 1,6859 | 0,50734 | 0,141 | -0,59 | -0,8 | -0,944 |  |  |  |  |  |  |
|  | *ptsH* | histidine-containing protein HPr | X276_20425 | 1,3449 | 0,06513 | 0,6988 | 0,194 | -1,22 | -1,079 |  |  |  |  |  |  |
|  | *hprK* | HPr kinase/phosphorylase | X276_19670 | 0,9277 | 1,22638 | 0,4851 | -0,91 | -1,06 | -0,667 |  |  |  |  |  |  |
|  | *manIIAB* | PTS enzyme EII AB subunit | X276_23245 | 0,7667 | -0,7216 | 1,4855 | -0,07 | -0,18 | -1,284 |  |  |  |  |  |  |
|  | *manIIC* | PTS enzyme EII C subunit | X276_23240 | 1,0946 | -0,6083 | 1,3616 | -0,34 | -0,35 | -1,157 |  |  |  |  |  |  |
|  | *manIID* | PTS enzyme EII D subunit | X276_23235 | 1,2952 | -0,6307 | 1,1756 | -0,39 | -0,31 | -1,137 |  |  |  |  |  |  |
|  | *manIID* | PTS enzyme EII D subunit | X276_02990 | 1,3877 | 0,41304 | 0,6174 | -0,19 | -1,07 | -1,156 |  |  |  |  |  |  |
|  | *manIIC* | PTS enzyme EII C subunit | X276_02985 | 1,406 | 0,52844 | 0,5849 | -0,4 | -1,05 | -1,064 |  |  |  |  |  |  |
|  | *manIIB* | PTS enzyme EII B subunit | X276_02980 | 1,2244 | 0,67697 | 0,6885 | -0,39 | -1,08 | -1,12 |  |  |  |  |  |  |
|  | *manIIAB* | PTS enzyme EII A subunit | X276_02975 | 1,4309 | 0,51173 | 0,5814 | -0,45 | -1,02 | -1,05 |  |  |  |  |  |  |
| **Glucose uptake via non-PTS** | | |  |  |  |  |  |  |  |  |  |  |  |  |  |
|  | *glcK* | glucokinase | X276_01140 | 0,1385 | 0,07376 | 0,0083 | 0,753 | 0,916 | -1,889 |  |  |  |  |  |  |
| **Glycolysis** | | |  |  |  |  |  |  |  |  |  |  |  |  |  |
|  | *pgi* | glucose-6-phosphate isomerase | X276_25070 | 1,2635 | 0,87857 | 0,5128 | -0,77 | -0,94 | -0,941 |  |  |  |  |  |  |
|  | *pfk* | 6-phosphofructokinase | X276_01370 | 0,9204 | 1,41787 | 0,1518 | -0,95 | -0,64 | -0,9 |  |  |  |  |  |  |
|  | *pfk* | ATP-dependent 6-phosphofructokinase | X276_21855 | 0,7064 | 1,36853 | -0,025 | 0,068 | -1,49 | -0,624 |  |  |  |  |  |  |
|  | *fba* | fructose-1,6-bisphosphate aldolase, class II | X276_17335 | 0,8225 | 1,37425 | 0,4048 | -0,77 | -0,94 | -0,89 |  |  |  |  |  |  |
|  | *tpi* | triose-phosphate isomerase | X276_23715 | 1,3433 | 1,10964 | -0,1161 | -1,06 | -0,55 | -0,73 |  |  |  |  |  |  |
|  | *gap* | type I glyceraldehyde-3-phosphate dehydrogenase | X276_23725 | 0,9998 | 1,38131 | 0,0661 | -1,04 | -0,62 | -0,784 |  |  |  |  |  |  |
|  | *pgk* | phosphoglycerate kinase | X276_23720 | 1,1739 | 1,25826 | -0,1212 | -1,15 | -0,47 | -0,691 |  |  |  |  |  |  |
|  | *gpm* | 2,3-bisphosphoglycerate-independent phosphoglycerate mutase | X276_23710 | 1,6157 | 0,77028 | -0,1055 | -0,93 | -0,74 | -0,611 |  |  |  |  |  |  |
|  | *eno* | enolase | X276_23700 | 1,7149 | 0,37491 | 0,2064 | -0,57 | -0,69 | -1,037 |  |  |  |  |  |  |
|  | *pyk* | pyruvate kinase | X276_01375 | 1,5452 | 0,91049 | -0,1964 | -0,81 | -0,59 | -0,865 |  |  |  |  |  |  |
|  | *pfo* | pyruvate:ferredoxin (flavodoxin) oxidoreductase | X276_03990 | 1,2765 | 1,12385 | 0,1053 | -0,82 | -0,88 | -0,803 |  |  |  |  |  |  |
|  | *ldh* | L-lactate dehydrogenase | X276_21775 | 1,8031 | 0,30701 | 0,0628 | -0,5 | -0,87 | -0,799 |  |  |  |  |  |  |
|  | *ldh* | L-lactate dehydrogenase | X276_05485 | -0,3489 | 0,53796 | 1,4563 | -1,56 | -0,18 | 0,0953 |  |  |  |  |  |  |
| **Acidogenesis** | | |  |  |  |  |  |  |  |  |  |  |  |  |  |
|  | *pta* | phosphate acetyltransferase | X276_20710 | 0,8341 | 1,32064 | 0,4683 | -1,01 | -0,91 | -0,707 |  |  |  |  |  |  |
|  | *ack* | acetate kinase | X276_20705 | 0,8283 | 1,23743 | 0,6103 | -0,97 | -0,93 | -0,77 |  |  |  |  |  |  |
|  | *ptb* | phosphate butyryltransferase | X276_25645 | 0,6975 | 0,94655 | 1,0423 | -0,61 | -0,98 | -1,096 |  |  |  |  |  |  |
|  | *buk* | butyrate kinase | X276_25640 | 1,2802 | 0,77205 | 0,618 | -0,87 | -0,95 | -0,843 |  |  |  |  |  |  |
| **Butyryl-CoA synthesis** | | |  |  |  |  |  |  |  |  |  |  |  |  |  |
|  | *thl* | acetyl-CoA C-acetyltransferase | X276_24605 | 1,7125 | 0,19835 | -0,0167 | 0,104 | -0,9 | -1,099 |  |  |  |  |  |  |
|  | *hbd* | 3-hydroxybutyryl-CoA dehydrogenase | X276_25200 | 1,741 | 0,38313 | -0,0319 | -0,21 | -0,87 | -1,006 |  |  |  |  |  |  |
|  | *crt* | crotonase | X276_25220 | 0,7527 | 0,94284 | 0,9894 | -0,79 | -1,22 | -0,681 |  |  |  |  |  |  |
|  | *etfA* | electron transfer flavoprotein subunit alpha/FixB family protein | X276_25205 | 1,8244 | 0,12784 | -0,2879 | 0,096 | -0,96 | -0,799 |  |  |  |  |  |  |
|  | *etfB* | electron transfer flavoprotein subunit beta/FixA family protein | X276_25210 | 1,7659 | 0,38281 | -0,094 | -0,23 | -0,98 | -0,844 |  |  |  |  |  |  |
|  | *bcd* | acyl-CoA dehydrogenase (butyrylCoA dehydrogenase) | X276_25215 | 1,6644 | 0,59074 | -0,0672 | -0,33 | -1,08 | -0,774 |  |  |  |  |  |  |
| **Solventogenesis** | | |  |  |  |  |  |  |  |  |  |  |  |  |  |
|  | *ctfA* | acetyl-CoA--acetoacetyl-CoA transferase subunit alpha | X276_06750 | -0,7192 | 1,9158 | -0,2415 | -0,81 | -0,28 | 0,1358 |  |  |  |  |  |  |
|  | *ctfB* | CoA transferase subunit B | X276_06745 | -0,8313 | 1,84443 | -0,1826 | -0,86 | -0,23 | 0,2684 |  |  |  |  |  |  |
|  | *adc* | acetoacetate decarboxylase | X276_06740 | -0,8554 | 1,86115 | -0,1571 | -0,82 | -0,24 | 0,2139 |  |  |  |  |  |  |
|  | *ald* | aldehyde dehydrogenase EutE; Provisional | X276_06755 | -0,7207 | 1,96191 | -0,1237 | -0,66 | -0,42 | -0,033 |  |  |  |  |  |  |
|  | *bdh* | iron-containing alcohol dehydrogenase | X276_18435 | 0,3252 | 1,27645 | 0,8967 | -0,33 | -1,12 | -1,044 |  |  |  |  |  |  |
|  | *bdh* | iron-containing alcohol dehydrogenase | X276_15910 | -0,7063 | 0,57931 | 1,7894 | -0,7 | -0,54 | -0,414 |  |  |  |  |  |  |
|  | *adh* | bifunctional acetaldehyde-CoA/alcohol dehydrogenase | X276_25300 | -0,3316 | -0,8824 | -0,8457 | 0,217 | 1,83 | 0,0123 |  |  |  |  |  |  |
|  |  |  |  | 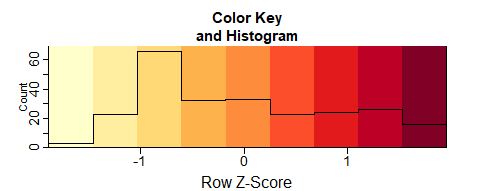   \|  \| \| --- \| |  |  |  |  |  |  |  |  |  |  |  |
|  |  |  |  |  |  |  |  |  |  |  |  |  |  |  |  |
|  |  |  |  |  |  |  |  |  |  |  |  |  |  |  |  |
|  |  |  |  |  |  |  |  |  |  |  |  |  |  |  |  |
|  |  |  |  |  |  |  |  |  |  |  |  |  |  |  |  |
|  |  |  |  |  |  |  |  |  |  |  |  |  |  |  |  |
|  |  |  |  |  |  |  |  |  |  |  |  |  |  |  |  |
|  |  |  |  |  |  |  |  |  |  |  |  |  |  |  |  |

| **Table A3: Granulose formation, sigma factors and Agr quorum sensing - RPKM values** | | | | | | | | | | | |  |  |  |  |
| --- | --- | --- | --- | --- | --- | --- | --- | --- | --- | --- | --- | --- | --- | --- | --- |
|  |  |  |  | Shocked ABE fermentation | | | | | | Standard ABE fermentation | | | | | |
|  | Gene | Putative physiological role | Locus tag | T_b_0 | T_b_1 | T_b_2 | T_b_3 | T_b_4 | T_b_5 | T1 | T2 | T3 | T4 | T5 | T6 |
| **Granulose formation** | | |  |  |  |  |  |  |  |  |  |  |  |  |  |
|  | *pgm* | phospho sugar mutase | X276_21930 | 720.51 | 737.31 | 773.48 | 824.38 | 758.93 | 727.69 | 429.08 | 922.80 | 811.98 | 388.47 | 271.56 | 291.60 |
|  | *glgD* | glucose-1-phosphate adenyl transferase | X276_01040 | 682.57 | 463.58 | 468.71 | 467.95 | 497.75 | 537.38 | 471.25 | 734.02 | 728.67 | 808.68 | 424.15 | 336.28 |
|  | *glgC* | glucose-1-phosphate adenyl transferase | X276_01035 | 883.61 | 639.97 | 655.79 | 631.75 | 675.77 | 745.85 | 559.36 | 964.53 | 913.77 | 908.02 | 525.71 | 414.67 |
|  | *amy* | glycoside hydrolase | X276_01030 | 522.46 | 434.81 | 440.95 | 450.04 | 464.14 | 420.40 | 371.89 | 464.13 | 401.38 | 366.27 | 222.87 | 219.14 |
|  | *glgP* | glycogen phosphorylase | X276_01025 | 658.34 | 614.81 | 647.91 | 635.30 | 607.05 | 568.13 | 455.58 | 645.74 | 580.00 | 483.25 | 355.54 | 342.73 |
|  | *glgA* | glycogen synthase | X276_01020 | 639.49 | 649.10 | 711.25 | 673.09 | 581.58 | 556.15 | 540.02 | 663.30 | 608.55 | 511.54 | 373.57 | 351.53 |
|  | *glgB* | branching enzyme | X276_01015 | 550.10 | 587.33 | 672.84 | 644.65 | 542.54 | 541.77 | 555.96 | 563.97 | 513.70 | 475.51 | 314.59 | 289.96 |
| **Sigma factors and regulators involved in sporulation** | | | |  |  |  |  |  |  |  |  |  |  |  |  |
|  | *sigA* | RNA polymerase sigma factor SigA | X276_22455 | 357.89 | 229.67 | 257.83 | 244.41 | 235.66 | 255.95 | 518.49 | 366.81 | 343.10 | 480.43 | 463.03 | 288.58 |
|  | *sigH* | RNA polymerase sigma factor SigH | X276_25990 | 410.20 | 362.36 | 453.58 | 474.03 | 444.99 | 461.86 | 311.85 | 399.22 | 385.07 | 298.08 | 279.81 | 290.23 |
|  | *sigF* | RNA polymerase sigma factor SigF | X276_22650 | 901.07 | 513.15 | 577.95 | 650.38 | 706.17 | 695.51 | 221.76 | 1145.10 | 1246.14 | 2066.63 | 801.40 | 668.35 |
|  | *sigE* | RNA polymerase sigma factor SigE | X276_20925 | 113.20 | 70.80 | 86.23 | 107.63 | 143.47 | 161.42 | 13.43 | 203.60 | 358.00 | 603.04 | 299.65 | 210.54 |
|  | *sigG* | RNA polymerase sigma factor SigG | X276_20920 | 159.86 | 91.73 | 105.92 | 120.36 | 149.67 | 205.58 | 65.69 | 256.16 | 671.56 | 1378.44 | 811.59 | 400.93 |
|  | *sigK* | RNA polymerase sigma factor SigK | X276_20950 | 135.00 | 132.38 | 143.21 | 137.43 | 109.96 | 98.74 | 335.21 | 113.48 | 204.41 | 1387.88 | 1677.35 | 926.42 |
|  | *spo0A* | sporulation transcription factor Spo0A | X276_18480 | 1767.28 | 1244.20 | 1223.11 | 1215.72 | 1317.03 | 1437.35 | 1166.23 | 2119.84 | 1704.73 | 2446.71 | 1123.43 | 928.35 |
|  | *abrB* | putative SigH repressor AbrB | X276_01205 | 538.16 | 991.58 | 808.83 | 528.53 | 273.15 | 301.98 | 1263.63 | 864.61 | 356.41 | 312.82 | 465.40 | 803.00 |
| **Agr quorum-sensing system** | | |  |  |  |  |  |  |  |  |  |  |  |  |  |
|  | *agrC* | sensor histidine kinase | X276_11845 | 28.57 | 11.24 | 9.48 | 8.38 | 11.09 | 10.07 | 24.58 | 31.75 | 39.83 | 19.21 | 12.19 | 9.55 |
|  | *agrD* | cyclic lactone autoinducer peptide | X276_27155 | 396.22 | 119.94 | 100.92 | 100.00 | 76.09 | 80.35 | 333.88 | 399.81 | 500.37 | 198.67 | 106.77 | 84.06 |
|  | *agrB* | membrane protein involved in AIP processing and secretion activity | X276_11840 | 331.36 | 113.95 | 98.89 | 70.50 | 71.73 | 76.04 | 325.76 | 297.84 | 248.59 | 110.82 | 73.23 | 59.55 |
|  | *agrA* | protein potentially involved in AIP signal transduction after autophosphorylation | X276_11835 | 216.58 | 77.42 | 68.78 | 42.23 | 38.64 | 41.37 | 291.29 | 205.11 | 175.76 | 92.33 | 50.35 | 41.15 |
|  |  |  |  |  |  |  |  |  |  |  |  |  |  |  |  |
|  | *smt* | class I SAM-dependent methyltransferase | X276_23505 | 4285.90 | 5005.45 | 3229.61 | 4391.60 | 5227.31 | 4444.14 | 13833.86 | 5359.59 | 4592.45 | 1291.81 | 1579.79 | 1288.86 |
|  | *agrB* | membrane protein involved in AIP processing and secretion activity | X276_23500 | 183.48 | 456.20 | 597.13 | 580.67 | 410.06 | 332.82 | 219.46 | 266.80 | 302.11 | 80.12 | 75.76 | 143.43 |
|  | *agrC* | sensor histidine kinase | X276_23495 | 45.06 | 120.45 | 130.95 | 112.22 | 78.01 | 71.14 | 66.75 | 61.56 | 36.72 | 15.22 | 15.24 | 25.46 |
|  | *agrB* | membrane protein involved in AIP processing and secretion activity | X276_23490 | 1233.65 | 1451.03 | 1579.14 | 1268.53 | 1063.52 | 888.92 | 1133.45 | 1129.20 | 1018.18 | 336.77 | 298.08 | 451.10 |
|  |  |  |  |  |  |  |  |  |  |  |  |  |  |  |  |
|  | *agrC* | sensor histidine kinase | X276_10565 | 31.23 | 5.03 | 3.89 | 5.21 | 4.01 | 5.86 | 36.40 | 33.17 | 48.70 | 45.03 | 18.71 | 10.20 |
|  | *agrD* | cyclic lactone autoinducer peptide | X276_27205 | 1806.67 | 712.45 | 485.21 | 340.79 | 358.31 | 321.59 | 2109.40 | 2954.92 | 6214.34 | 1613.05 | 581.55 | 330.65 |
|  | *agrA* | protein potentially involved in AIP signal transduction after autophosphorylation | X276_10560 | 198.86 | 27.99 | 27.65 | 35.88 | 37.78 | 34.44 | 233.27 | 175.30 | 216.28 | 107.89 | 61.94 | 45.92 |
|  | *agrB* | membrane protein involved in AIP processing and secretion activity | X276_10555 | 189.43 | 35.32 | 44.56 | 47.12 | 51.30 | 46.35 | 192.61 | 167.29 | 195.25 | 89.27 | 48.81 | 39.43 |
|  |  |  |  |  |  |  |  |  |  |  |  |  |  |  |  |
|  |  |  |  |  |  |  |  |  |  |  |  |  |  |  |  |
| **Table A3 additional information: Granulose formation, sigma factors, and Agr quorum sensing - Heatmap (standard ABE fermentation)** | | | | | | | | | | | | | | | |
|  |  |  |  | Standard ABE fermentation | | | | | |  |  |  |  |  |  |
|  | Gene | Putative physiological role | Locus tag | T1 | T2 | T3 | T4 | T5 | T6 |  |  |  |  |  |  |
| **Granulose formation** | | |  |  |  |  |  |  |  | 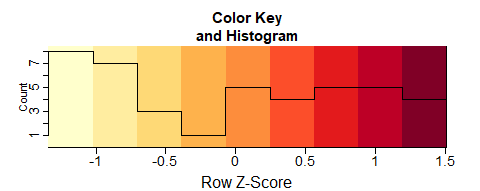   \|  \| \| --- \| |  |  |  |  |  |
|  | *pgm* | phospho sugar mutase | X276_21930 | 0,1147 | 1,5037 | 0,832 | -0,7184 | -0,9597 | -0,7723 |  |  |  |  |  |  |
|  | *glgD* | glucose-1-phosphate adenyl transferase | X276_01040 | 0,2071 | 1,1184 | 0,6599 | 0,4734 | -1,1205 | -1,3383 |  |  |  |  |  |  |
|  | *glgC* | glucose-1-phosphate adenyl transferase | X276_01035 | 0,0799 | 1,3549 | 0,7015 | 0,1527 | -1,037 | -1,2519 |  |  |  |  |  |  |
|  | *amy* | glycoside hydrolase | X276_01030 | 1,1016 | 1,1312 | 0,2767 | -0,3801 | -1,1611 | -0,9684 |  |  |  |  |  |  |
|  | *glgP* | glycogen phosphorylase | X276_01025 | 0,7671 | 1,3508 | 0,4718 | -0,5987 | -1,1046 | -0,8865 |  |  |  |  |  |  |
|  | *glgA* | glycogen synthase | X276_01020 | 1,1281 | 1,0998 | 0,3711 | -0,5872 | -1,0811 | -0,9308 |  |  |  |  |  |  |
|  | *glgB* | branching enzyme | X276_01015 | 1,5137 | 0,7495 | 0,1348 | -0,4265 | -1,0266 | -0,945 |  |  |  |  |  |  |
| **Sigma factors and regulators involved in sporulation** | | | |  |  |  |  |  |  |  |  |  |  |  |  |
|  | *sigA* | RNA polymerase sigma factor SigA | X276_22455 | 1,9264 | -0,2305 | -0,6633 | -0,1879 | 0,02687 | -0,8716 |  |  |  |  |  |  |
|  | *sigH* | RNA polymerase sigma factor SigH | X276_25990 | 0,9867 | 1,1469 | 0,4419 | -1,1469 | -1,0142 | -0,4145 | 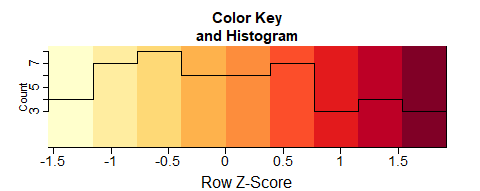   \|  \| \| --- \| |  |  |  |  |  |
|  | *sigF* | RNA polymerase sigma factor SigF | X276_22650 | -1,37 | 0,494 | 0,4406 | 1,4665 | -0,478 | -0,5532 |  |  |  |  |  |  |
|  | *sigE* | RNA polymerase sigma factor SigE | X276_20925 | -1,5417 | -0,2804 | 0,5108 | 1,4858 | 0,08707 | -0,2615 |  |  |  |  |  |  |
|  | *sigG* | RNA polymerase sigma factor SigG | X276_20920 | -1,2447 | -0,7417 | 0,2439 | 1,562 | 0,52888 | -0,3484 |  |  |  |  |  |  |
|  | *sigK* | RNA polymerase sigma factor SigK | X276_20950 | -0,5111 | -1,0763 | -0,9466 | 0,6914 | 1,42566 | 0,41693 |  |  |  |  |  |  |
|  | *spo0A* | sporulation transcription factor Spo0A | X276_18480 | -0,0758 | 1,317 | 0,1179 | 0,8694 | -1,0559 | -1,1726 |  |  |  |  |  |  |
|  | *abrB* | putative SigH repressor AbrB | X276_01205 | 1,75 | 0,3354 | -0,7604 | -0,9238 | -0,596 | 0,19479 |  |  |  |  |  |  |
| **Agr quorum-sensing system** | | |  |  |  |  |  |  |  |  |  |  |  |  |  |
|  | *agrC* | sensor histidine kinase | X276_11845 | 0,6983 | 0,8064 | 1,1685 | -0,6206 | -0,9775 | -1,0752 |  |  |  |  |  |  |
|  | *agrD* | cyclic lactone autoinducer peptide | X276_27155 | 0,8371 | 0,7655 | 1,0935 | -0,6489 | -0,9969 | -1,0501 |  |  |  |  |  |  |
|  | *agrB* | membrane protein involved in AIP processing and secretion activity | X276_11840 | 1,4945 | 0,7593 | 0,2579 | -0,721 | -0,8783 | -0,9124 |  |  |  |  |  |  |
|  | *agrA* | protein potentially involved in AIP signal transduction after autophosporylation | X276_11835 | 1,7266 | 0,4687 | 0,0981 | -0,6089 | -0,8296 | -0,8549 |  |  |  |  |  |  |
|  |  |  |  |  |  |  |  |  |  | 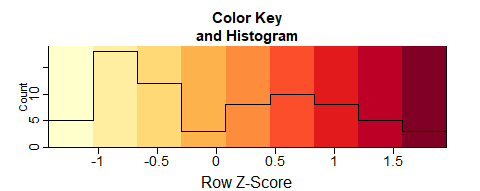 | | | | | |
|  | *smt* | class I SAM-dependent methyltransferase | X276_23505 | 1,9553 | 0,0489 | -0,1457 | -0,6542 | -0,5942 | -0,61 |  |  |  |  |  |  |
|  | *agrB* | membrane protein involved in AIP processing and secretion activity | X276_23500 | 0,8901 | 0,8388 | 0,9022 | -1,1466 | -1,1119 | -0,3726 |  |  |  |  |  |  |
|  | *agrC* | sensor histidine kinase | X276_23495 | 1,5755 | 0,8267 | -0,1588 | -0,9207 | -0,8693 | -0,4534 |  |  |  |  |  |  |
|  | *agrB* | membrane protein involved in AIP processing and secretion activity | X276_23490 | 1,3864 | 0,7989 | 0,382 | -0,9788 | -0,9871 | -0,6013 |  |  |  |  |  |  |
|  |  |  |  |  |  |  |  |  |  |  |  |  |  |  |  |
|  | *agrC* | sensor histidine kinase | X276_10565 | 0,9984 | 0,1751 | 0,9402 | 0,2984 | -0,9935 | -1,4186 |  |  |  |  |  |  |
|  | *agrD* | cyclic lactone autoinducer peptide | X276_27205 | 0,2053 | 0,3781 | 1,7132 | -0,4853 | -0,8613 | -0,95 |  |  |  |  |  |  |
|  | *agrA* | protein potentially involved in AIP signal transduction after autophosporylation | X276_10560 | 1,5471 | 0,361 | 0,5764 | -0,5938 | -0,9016 | -0,9892 |  |  |  |  |  |  |
|  | *agrB* | membrane protein involved in AIP processing and secretion activity | X276_10555 | 1,4023 | 0,5398 | 0,6499 | -0,6342 | -0,9582 | -0,9997 |  |  |  |  |  |  |

| **Table A4: Polyketide biosynthesis - RPKM values** | | | | | | |  |  |  |  |  |  |  |  |  |
| --- | --- | --- | --- | --- | --- | --- | --- | --- | --- | --- | --- | --- | --- | --- | --- |
| **Cluster A** | |  |  | Shocked ABE fermentation | | | | | | Standard ABE fermentation | | | | | |
|  | Gene | Putative physiological role | Locus tag | T_b_0 | T_b_1 | T_b_2 | T_b_3 | T_b_4 | T_b_5 | T1 | T2 | T3 | T4 | T5 | T6 |
|  | *nrps* | non-ribosomal peptide synthase | X276_04700 | 302.71 | 69.55 | 70.72 | 78.29 | 71.39 | 58.62 | 576.60 | 184.56 | 290.70 | 330.25 | 78.23 | 34.65 |
|  | *acpm* | ACP S-malonyltransferase | X276_04695 | 337.33 | 38.22 | 43.97 | 41.63 | 36.45 | 29.82 | 575.23 | 188.22 | 336.64 | 383.68 | 81.47 | 18.73 |
|  | *nrps* | non-ribosomal peptide synthetase | X276_04690 | 446.36 | 42.87 | 47.39 | 51.25 | 44.58 | 35.55 | 575.71 | 223.00 | 420.27 | 303.39 | 99.17 | 26.67 |
|  | *tes* | thioesterase | X276_04685 | 464.86 | 37.58 | 38.47 | 36.99 | 50.14 | 46.94 | 371.83 | 296.21 | 475.95 | 331.11 | 84.45 | 28.58 |
| **Cluster C** | |  |  |  |  |  |  |  |  |  |  |  |  |  |  |
|  | *baa* | benzoate-CoA ligase | X276_10460 | 100.38 | 86.08 | 33.03 | 33.26 | 50.02 | 55.26 | 25.93 | 100.97 | 81.46 | 39.42 | 49.83 | 46.94 |
|  | *acp* | acyl carrier protein | X276_10455 | 103.26 | 64.08 | 14.53 | 8.50 | 22.66 | 42.63 | 26.37 | 96.86 | 78.90 | 37.00 | 47.76 | 46.01 |
|  | *abh* | alpha/beta hydrolase | X276_10450 | 81.61 | 61.93 | 16.49 | 21.27 | 30.16 | 31.57 | 20.83 | 80.80 | 61.88 | 27.33 | 39.55 | 34.28 |
|  | *ppt* | 4'-phosphopantetheinyl transferase superfamily protein | X276_10445 | 41.77 | 37.42 | 13.82 | 14.28 | 27.11 | 20.37 | 11.96 | 36.30 | 28.64 | 13.52 | 20.56 | 20.05 |
|  | *aes* | acetyl esterase | X276_10440 | 92.02 | 73.36 | 38.00 | 31.86 | 48.92 | 55.03 | 25.13 | 84.89 | 59.67 | 30.89 | 46.37 | 46.74 |
|  | *pks* | undefined polyketide synthase | X276_27625 | 60.98 | 45.84 | 21.76 | 22.17 | 31.98 | 36.36 | 17.04 | 55.46 | 43.31 | 17.67 | 31.73 | 32.61 |
|  | *acp* | acyl carrier protein | X276_10425 | 55.17 | 28.03 | 13.37 | 10.35 | 25.41 | 26.77 | 16.53 | 59.25 | 42.83 | 20.46 | 34.53 | 32.14 |
|  | *smt* | class I SAM methyltransferase | X276_10420 | 56.94 | 39.40 | 16.80 | 17.84 | 30.63 | 26.88 | 17.99 | 57.10 | 43.88 | 18.81 | 28.76 | 23.25 |
|  | *amp* | AMP-binding protein | X276_10415 | 71.12 | 38.71 | 21.81 | 22.46 | 38.97 | 37.66 | 20.66 | 65.54 | 50.29 | 18.45 | 29.59 | 29.12 |
|  | *hypo* | hypothetical protein | X276_10410 | 85.31 | 53.74 | 32.26 | 38.71 | 40.81 | 41.74 | 21.54 | 69.31 | 55.77 | 23.96 | 34.43 | 34.70 |
|  | *pks* | type I PKS synthase | X276_27215 | 66.75 | 32.73 | 24.21 | 25.53 | 35.68 | 36.86 | 20.69 | 58.61 | 47.88 | 24.64 | 37.37 | 30.96 |
|  | *pks* | type I PKS synthase | X276_10400 | 85.99 | 37.17 | 30.70 | 38.85 | 48.78 | 46.34 | 28.11 | 75.23 | 60.11 | 25.87 | 40.88 | 38.68 |
|  | *pks* | type I PKS synthase | X276_10395 | 104.22 | 29.06 | 25.92 | 31.76 | 47.05 | 45.42 | 39.27 | 82.84 | 70.76 | 29.75 | 49.17 | 48.93 |
|  | *ech* | enoyl-CoA hydratase | X276_10390 | 171.29 | 25.42 | 24.45 | 37.55 | 60.55 | 60.53 | 58.02 | 91.26 | 84.73 | 35.07 | 71.46 | 80.12 |
|  | *hmg* | hydroxymethylglutaryl-CoA synthase family protein | X276_10385 | 178.25 | 26.95 | 25.84 | 41.41 | 55.46 | 65.75 | 68.02 | 103.70 | 96.43 | 41.27 | 76.48 | 87.17 |
|  | *acp* | acyl carrier protein | X276_10380 | 160.78 | 13.08 | 13.85 | 18.59 | 44.75 | 41.13 | 72.30 | 108.88 | 111.18 | 45.34 | 84.69 | 81.13 |
|  | *ech* | enoyl-CoA hydratase/isomerase | X276_10375 | 111.96 | 14.70 | 12.04 | 21.89 | 26.32 | 36.39 | 42.13 | 58.32 | 55.08 | 24.28 | 47.12 | 45.66 |
|  | *hmg* | hydroxymethylglutaryl-CoA synthase family protein | X276_10370 | 194.10 | 24.15 | 27.80 | 35.37 | 60.65 | 66.87 | 82.98 | 127.11 | 118.36 | 50.88 | 91.04 | 100.42 |
|  | *pks* | polyketide beta-ketoacyl:ACP synthase | X276_10365 | 194.28 | 28.69 | 23.84 | 34.36 | 66.37 | 59.43 | 84.29 | 118.61 | 114.86 | 53.33 | 89.94 | 92.89 |
|  | *acpm* | ACP S-malonyltransferase | X276_10360 | 213.91 | 22.08 | 16.33 | 29.92 | 62.98 | 62.71 | 88.75 | 135.84 | 137.69 | 65.93 | 94.96 | 98.54 |
|  | *hbla* | beta-lactamase domain protein | X276_10355 | 441.35 | 98.43 | 93.32 | 122.59 | 186.63 | 197.84 | 229.75 | 321.40 | 296.76 | 144.02 | 165.65 | 190.68 |
|  | *loaP* | antiterminator LoaP | X276_10350 | 88.25 | 44.02 | 37.13 | 44.04 | 64.97 | 58.29 | 65.30 | 82.48 | 76.60 | 60.16 | 43.00 | 58.24 |
|  | *purH* | phosphoribosylaminoimidazolecarboxamide formyltransferase | X276_10345 | 156.02 | 87.23 | 84.42 | 86.28 | 137.46 | 160.83 | 112.66 | 158.30 | 138.51 | 176.76 | 110.89 | 119.81 |
|  | *dhbB* | isochorismatase | X276_10335 | 451.38 | 277.18 | 198.73 | 210.66 | 305.84 | 335.67 | 495.72 | 371.98 | 272.44 | 185.30 | 198.97 | 283.49 |
|  | *ics* | isochorismate synthase | X276_10330 | 505.22 | 338.32 | 250.25 | 253.75 | 355.54 | 419.73 | 491.34 | 391.77 | 306.36 | 186.33 | 189.51 | 295.67 |
|  | *entA* | 2,3-dihydro-2,3-dihydroxybenzoate dehydrogenase | X276_10325 | 670.93 | 451.14 | 314.08 | 312.13 | 449.76 | 527.74 | 612.31 | 527.29 | 458.25 | 276.51 | 266.20 | 408.25 |
|  | *amp* | AMP-binding protein | X276_10320 | 750.86 | 457.60 | 331.52 | 357.26 | 550.23 | 569.11 | 568.92 | 533.58 | 418.08 | 257.54 | 260.62 | 387.52 |
|  | *ppt* | 4'-phosphopantetheinyl transferase | X276_10315 | 1004.48 | 619.42 | 459.45 | 478.45 | 768.92 | 731.05 | 789.30 | 789.02 | 624.63 | 403.75 | 405.05 | 537.56 |
| **Cluster B** | |  |  |  |  |  |  |  |  |  |  |  |  |  |  |
|  | *pks* | putative PKS synthase | X276_07725 | 4.53 | 3.03 | 2.70 | 3.67 | 3.86 | 4.06 | 3.72 | 4.37 | 4.59 | 9.14 | 11.71 | 9.99 |
|  | *sdro* | SDR family NAD(P)-dependent oxidoreductase | X276_07720 | 5.66 | 3.50 | 4.12 | 4.83 | 4.21 | 5.09 | 4.78 | 4.23 | 5.68 | 13.98 | 18.64 | 16.02 |
|  | *mtd* | methyltransferase domain-containing protein | X276_27285 | 7.53 | 3.50 | 4.28 | 4.70 | 5.14 | 5.67 | 6.93 | 5.77 | 6.70 | 19.54 | 26.35 | 25.94 |
|  | *nrps* | non-ribosomal peptide synthase | X276_07705 | 13.06 | 3.39 | 3.39 | 3.94 | 5.57 | 4.64 | 8.25 | 5.06 | 7.48 | 18.92 | 20.53 | 16.10 |
|  | *nrps* | non-ribosomal peptide synthase | X276_07700 | 56.13 | 7.18 | 14.15 | 27.96 | 84.52 | 19.21 | 39.73 | 16.69 | 26.37 | 43.09 | 20.44 | 26.89 |

| **Table A5: Heat-shock proteins - RPKM values** | | | | | |  |  |  |  |  |  |  |  |  |
| --- | --- | --- | --- | --- | --- | --- | --- | --- | --- | --- | --- | --- | --- | --- |
|  |  |  | Shocked ABE fermentation | | | | | | Standard ABE fermentation | | | | | |
| Gene | Putative physiological role | Locus tag | T_b_0 | T_b_1 | T_b_2 | T_b_3 | T_b_4 | T_b_5 | T1 | T2 | T3 | T4 | T5 | T6 |
| *radA* | DNA repair protein RadA | X276_26035 | 203.89 | 540.51 | 462.75 | 376.23 | 304.84 | 328.63 | 251.61 | 318.56 | 139.09 | 158.56 | 200.91 | 197.37 |
| *htpG* | molecular chaperone HtpG | X276_05050 | 256.84 | 748.49 | 910.31 | 1007.22 | 858.13 | 738.93 | 264.45 | 400.34 | 459.99 | 403.76 | 291.03 | 323.46 |
| *dnaJ* | molecular chaperone DnaJ | X276_22565 | 723.53 | 4998.37 | 4438.65 | 3412.17 | 2898.88 | 3028.60 | 912.05 | 1621.30 | 375.58 | 288.71 | 1021.21 | 1580.42 |
| *dnaK* | molecular chaperone DnaK | X276_22570 | 845.28 | 10470.12 | 7495.07 | 5011.15 | 3870.82 | 4168.83 | 1354.21 | 2290.48 | 541.32 | 361.88 | 1570.08 | 3008.98 |
| *grpE* | heat shock protein GrpE | X276_22575 | 275.46 | 4009.36 | 2733.94 | 2299.62 | 2002.31 | 1782.62 | 409.69 | 545.80 | 255.74 | 226.69 | 717.03 | 1155.97 |
| *groEL* | chaperonin GroEL | X276_25130 | 3810.34 | 26881.98 | 27664.05 | 24175.71 | 16265.48 | 16589.91 | 2856.30 | 9546.27 | 2480.55 | 864.18 | 3284.41 | 5537.96 |
| *groES* | chaperonin GroES | X276_25135 | 2667.43 | 20154.95 | 20807.22 | 19268.98 | 12499.79 | 12578.82 | 1891.37 | 6490.16 | 2013.05 | 686.56 | 2489.76 | 4322.58 |
| *hrcA* | heat-inducible transcription repressor | X276_22580 | 242.72 | 2836.79 | 2482.11 | 2422.53 | 2246.60 | 1879.69 | 197.60 | 309.30 | 200.47 | 166.73 | 484.09 | 913.69 |
| *sigI* | RNA polymerase sigma factor I | X276_17720 | 5352.80 | 4034.36 | 4520.67 | 4247.65 | 4319.12 | 4649.12 | 4720.24 | 5453.46 | 7152.16 | 9198.09 | 7674.55 | 7022.06 |
| *ctsR* | transcriptional regulator CtsR | X276_26065 | 140.70 | 1144.26 | 953.06 | 610.42 | 424.50 | 387.32 | 305.86 | 231.52 | 108.57 | 110.39 | 151.73 | 192.32 |
| *clpP* | ATP-dependent Clp protease proteolytic subunit | X276_19860 | 896.50 | 846.75 | 859.14 | 926.15 | 1031.03 | 1084.11 | 824.73 | 1006.72 | 1135.43 | 1224.21 | 1201.87 | 1002.30 |
| *clpX* | ATP-dependent Clp protease ATP-binding subunit | X276_19855 | 1200.70 | 1129.04 | 1186.22 | 1299.50 | 1370.29 | 1386.47 | 1212.53 | 1322.96 | 1555.88 | 1821.70 | 1868.80 | 1602.13 |
| *ibpA* | molecular chaperone IbpA | X276_05235 | 3401.96 | 30092.91 | 44046.76 | 33703.47 | 15816.76 | 12016.14 | 1742.62 | 9480.28 | 2665.03 | 590.06 | 1048.40 | 2440.34 |
| *asp23* | Asp23/Gls24 family envelope stress response protein | X276_18540 | 919.66 | 705.40 | 653.88 | 670.74 | 690.49 | 779.57 | 940.77 | 1215.45 | 1978.19 | 3022.18 | 2236.39 | 1167.31 |
| *tig* | trigger factor | X276_19865 | 991.51 | 635.74 | 636.10 | 821.06 | 1075.65 | 1236.19 | 1215.71 | 1195.94 | 1176.74 | 1304.89 | 1010.99 | 781.35 |
| *cspA* | cold-shock protein | X276_11750 | 673.34 | 614.85 | 530.60 | 528.69 | 528.38 | 595.57 | 796.41 | 792.59 | 997.92 | 1726.25 | 1241.53 | 673.96 |
|  |  |  |  |  |  |  |  |  |  |  |  |  |  |  |
|  |  |  |  |  |  |  |  |  |  |  |  |  |  |  |
| **Table A5 additional information: Heat-shock proteins - Heatmap (standard ABE fermentation)** | | | | | | | | | | | | | | |
|  |  |  | Standard ABE fermentation | | | | | |  |  |  |  |  |  |
| Gene | Putative physiological role | Locus tag | T1 | T2 | T3 | T4 | T5 | T6 |  |  |  |  |  |  |
| *radA* | DNA repair protein RadA | X276_26035 | 1,19 | 1,2606502 | -0,93921 | -0,96698 | -0,39319 | -0,15127 |  |  |  |  |  |  |
| *htpG* | molecular chaperone HtpG | X276_05050 | -0,1535 | 1,0296161 | 1,27471 | -0,40673 | -1,42208 | -0,32201 |  |  |  |  |  |  |
| *dnaJ* | molecular chaperone DnaJ | X276_22565 | 0,29548 | 1,1138901 | -1,04749 | -1,24045 | -0,13082 | 1,009391 |  |  |  |  |  |  |
| *dnaK* | molecular chaperone DnaK | X276_22570 | 0,16676 | 0,7537979 | -0,98926 | -1,19045 | -0,14999 | 1,409138 |  |  |  |  |  |  |
| *grpE* | heat shock protein GrpE | X276_22575 | -0,08223 | 0,026218 | -0,88616 | -1,04584 | 0,239881 | 1,748127 | 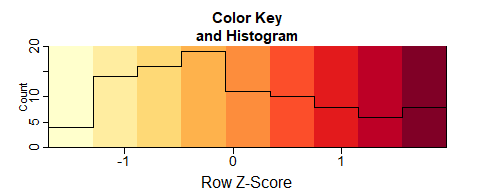   \|  \| \| --- \| |  |  |  |  |  |
| *groEL* | chaperonin GroEL | X276_25130 | -0,15771 | 1,7616149 | -0,58056 | -1,08568 | -0,38964 | 0,45197 |  |  |  |  |  |  |
| *groES* | chaperonin GroES | X276_25135 | -0,2721 | 1,6786695 | -0,52778 | -1,13786 | -0,37107 | 0,630134 |  |  |  |  |  |  |
| *hrcA* | heat-inducible transcription repressor | X276_22580 | -0,42011 | -0,194088 | -0,64953 | -0,83417 | 0,19235 | 1,905547 |  |  |  |  |  |  |
| *sigI* | RNA polymerase sigma factor I | X276_17720 | -0,87439 | -1,490519 | 0,07388 | 1,078418 | 0,371621 | 0,840986 |  |  |  |  |  |  |
| *ctsR* | transcriptional regulator CtsR | X276_26065 | 1,76253 | 0,4121309 | -0,79994 | -0,91349 | -0,49792 | 0,03669 |  |  |  |  |  |  |
| *clpP* | ATP-dependent Clp protease proteolytic subunit | X276_19860 | 0,5589 | 0,0392071 | 0,663893 | -1,70065 | 1,01208 | -0,57343 |  |  |  |  |  |  |
| *clpX* | ATP-dependent Clp protease ATP-binding subunit | X276_19855 | 0,27539 | -1,346467 | -0,58701 | -0,54048 | 1,269041 | 0,92953 |  |  |  |  |  |  |
| *ibpA* | molecular chaperone IbpA | X276_05235 | -0,24151 | 1,9757914 | -0,16957 | -0,76555 | -0,62737 | -0,1718 |  |  |  |  |  |  |
| *asp23* | Asp23/Gls24 family envelope stress response protein | X276_18540 | -0,86148 | -0,77224 | 0,368393 | 1,502111 | 0,669458 | -0,90624 |  |  |  |  |  |  |
| *tig* | trigger factor | X276_19865 | 1,67492 | 0,48745 | -0,02884 | -0,22423 | -0,75068 | -1,15862 |  |  |  |  |  |  |
| *cspA* | cold-shock protein | X276_11750 | 0,11798 | -0,705398 | -0,25354 | 1,607373 | 0,496247 | -1,26266 |  |  |  |  |  |  |

| **Table A6: Fatty acids synthesis - RPKM values** | | | | | | | |  |  |  |  |  |  |  |
| --- | --- | --- | --- | --- | --- | --- | --- | --- | --- | --- | --- | --- | --- | --- |
| **Gene** | **Putative physiological role** |  |  |  |  |  |  |  |  |  |  |  |  |  |
| *fabH* | beta-ketoacyl-ACP synthase (III) | | |  |  |  |  |  |  |  |  |  |  |  |
| *fabK* | enoyl-ACP-reductase | |  |  |  |  |  |  |  |  |  |  |  |  |
| *fabD* | malonyl-CoA-ACP transacylase | | |  |  |  |  |  |  |  |  |  |  |  |
| *fabG* | beta-ketoacyl ACP reductase | | |  |  |  |  |  |  |  |  |  |  |  |
| *fabF* | beta-ketoacyl-ACP synthase | | |  |  |  |  |  |  |  |  |  |  |  |
| *accB* | acetyl-CoA carboxylase subunit B | | |  |  |  |  |  |  |  |  |  |  |  |
| *fabZ* | beta-hydroxyacyl-ACP dehydratase | | | |  |  |  |  |  |  |  |  |  |  |
| *accC* | acetyl-CoA carboxylase subunit C | | |  |  |  |  |  |  |  |  |  |  |  |
| *accD* | acetyl-CoA carboxylase subunit D | | |  |  |  |  |  |  |  |  |  |  |  |
| *accA* | acetyl-CoA carboxylase subunit A | | |  |  |  |  |  |  |  |  |  |  |  |
| *acp* | acyl carrier protein | |  |  |  |  |  |  |  |  |  |  |  |  |
| *cfa* | cyclopropane fatty acid synthase | | |  |  |  |  |  |  |  |  |  |  |  |
|  |  |  |  |  |  |  |  |  |  |  |  |  |  |  |
|  |  |  |  |  |  |  |  |  |  |  |  |  |  |  |
|  |  |  |  |  |  |  |  |  |  |  |  |  |  |  |
|  |  | Shocked ABE fermentation | | | | | | Standard ABE fermentation | | | | | |  |
| Gene | Locus tag | T_b_0 | T_b_1 | T_b_2 | T_b_3 | T_b_4 | T_b_5 | T1 | T2 | T3 | T4 | T5 | T6 |  |
| *fabH* | X276_21490 | 176.60 | 101.75 | 90.19 | 144.67 | 195.56 | 214.19 | 137.83 | 173.78 | 306.38 | 421.61 | 194.50 | 166.25 |  |
| *fabK* | X276_21485 | 363.51 | 210.84 | 195.26 | 265.14 | 375.60 | 382.40 | 387.48 | 384.78 | 542.73 | 740.89 | 382.45 | 332.45 |  |
| *fabD* | X276_21480 | 279.14 | 170.10 | 154.85 | 231.08 | 310.04 | 326.68 | 329.67 | 291.96 | 428.76 | 647.63 | 347.36 | 278.94 |  |
| *fabG* | X276_21475 | 299.78 | 158.70 | 138.90 | 174.14 | 298.05 | 312.37 | 358.35 | 307.38 | 424.01 | 613.99 | 317.17 | 239.05 |  |
| *fabF* | X276_21470 | 462.36 | 215.39 | 200.13 | 276.14 | 415.89 | 440.29 | 554.88 | 424.64 | 578.77 | 842.73 | 440.05 | 346.97 |  |
| *accB* | X276_21465 | 287.81 | 173.28 | 154.36 | 198.55 | 255.29 | 268.24 | 366.77 | 289.00 | 352.84 | 449.06 | 297.98 | 262.95 |  |
| *fabZ* | X276_21460 | 349.07 | 223.20 | 199.00 | 250.43 | 310.25 | 320.69 | 439.61 | 355.47 | 437.62 | 552.77 | 342.66 | 268.02 |  |
| *accC* | X276_21455 | 345.04 | 227.44 | 223.87 | 284.78 | 346.24 | 334.16 | 402.50 | 328.84 | 400.75 | 556.57 | 360.39 | 287.84 |  |
| *accD* | X276_21450 | 315.33 | 218.55 | 211.00 | 269.39 | 322.26 | 319.29 | 359.40 | 281.50 | 371.48 | 582.78 | 363.53 | 283.66 |  |
| *accA* | X276_21445 | 266.99 | 171.89 | 182.05 | 226.26 | 282.63 | 295.24 | 293.36 | 229.15 | 310.78 | 546.94 | 341.16 | 256.66 |  |
| *acp* | X276_20685 | 1330.24 | 1063.80 | 1034.85 | 1361.60 | 1413.45 | 1424.45 | 2619.15 | 1787.95 | 2186.85 | 3282.32 | 2091.24 | 1582.81 |  |
| *cfa* | X276_00620 | 1139.59 | 2031.44 | 1914.01 | 1831.36 | 1722.88 | 1557.28 | 718.22 | 1471.88 | 734.56 | 356.74 | 631.32 | 394.65 |  |
|  |  |  |  |  |  |  |  |  |  |  |  |  |  |  |
| **Table A6 additional information: Fatty acids synthesis - Heatmap (standard ABE fermentation)** | | | | | | | | | | |  |  |  |  |
|  |  | Standard ABE fermentation | | | | | |  |  |  |  |  |  |  |
| Gene | Locus tag | T1 | T2 | T3 | T4 | T5 | T6 |  |  |  |  |  |  |  |
| *fabH* | X276_21490 | -0,60190923 | -0,5580737 | 0,8739 | 1,6301 | -0,639189 | -0,7048 | 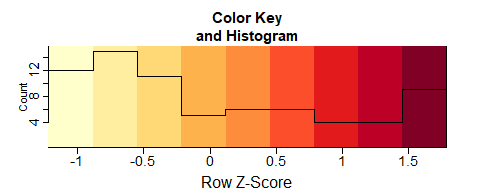 | | | | | | |
| *fabK* | X276_21485 | 0,476366918 | -0,472616 | 0,56057 | 1,47097 | -1,001742 | -1,0335 |  |  |  |  |  |  |  |
| *fabD* | X276_21480 | 0,530727363 | -0,7758776 | 0,27941 | 1,62102 | -0,68517 | -0,9701 |  |  |  |  |  |  |  |
| *fabG* | X276_21475 | 0,929433545 | -0,4787592 | 0,28815 | 1,29502 | -0,818723 | -1,2151 |  |  |  |  |  |  |  |
| *fabF* | X276_21470 | 1,275853668 | -0,5384436 | 0,13947 | 1,06709 | -0,84039 | -1,1036 |  |  |  |  |  |  |  |
| *accB* | X276_21465 | 1,788019659 | -0,444356 | -0,0457 | 0,40067 | -0,860247 | -0,8384 |  |  |  |  |  |  |  |
| *fabZ* | X276_21460 | 1,618589711 | -0,2620617 | 0,1315 | 0,50624 | -0,840017 | -1,1542 |  |  |  |  |  |  |  |
| *accC* | X276_21455 | 1,571451532 | -0,508537 | -0,0935 | 0,80032 | -0,716427 | -1,0533 |  |  |  |  |  |  |  |
| *accD* | X276_21450 | 1,192474457 | -0,8897072 | -0,1989 | 1,29366 | -0,478801 | -0,9187 |  |  |  |  |  |  |  |
| *accA* | X276_21445 | 0,758220768 | -1,0650181 | -0,3624 | 1,6023 | -0,186563 | -0,7465 |  |  |  |  |  |  |  |
| *acp* | X276_20685 | 1,732101449 | -0,5868793 | -0,2827 | 0,62551 | -0,547645 | -0,9404 |  |  |  |  |  |  |  |
| *cfa* | X276_00620 | 0,437815589 | 1,76740318 | -0,1023 | -1 | -0,369846 | -0,7331 |  |  |  |  |  |  |  |
|  |  |  |  |  |  |  |  |  |  |  |  |  |  |  |
|  |  |  |  |  |  |  |  |  |  |  |  |  |  |  |
| **Table A6 additional information: Fatty acids synthesis - Putative operon organization** | | | | | | | | | |  |  |  |  |  |
|  |  | Genome2D | Correlation matrix | | | | | | | | | | | |
| Gene | Locus tag |  | X276_21490 | _21485 | _21480 | _21475 | _21470 | _21465 | _21460 | _21455 | _21450 | _21445 | _20685 | _00620 |
| *fabH* | X276_21490 | operon_0754 | 1.00 | 0.91 | 0.90 | 0.82 | 0.75 | 0.55 | 0.57 | 0.61 | 0.72 | 0.77 | 0.50 | -0.71 |
| *fabK* | X276_21485 | operon_0755 | 0.91 | 1.00 | 0.98 | 0.98 | 0.95 | 0.84 | 0.85 | 0.87 | 0.89 | 0.87 | 0.78 | -0.77 |
| *fabD* | X276_21480 | operon_0755 | 0.90 | 0.98 | 1.00 | 0.97 | 0.94 | 0.83 | 0.82 | 0.87 | 0.93 | 0.94 | 0.81 | -0.80 |
| *fabG* | X276_21475 | operon_0756 | 0.82 | 0.98 | 0.97 | 1.00 | 0.99 | 0.91 | 0.92 | 0.93 | 0.93 | 0.89 | 0.84 | -0.74 |
| *fabF* | X276_21470 | operon_0756 | 0.75 | 0.95 | 0.94 | 0.99 | 1.00 | 0.95 | 0.95 | 0.97 | 0.94 | 0.88 | 0.87 | -0.72 |
| *accB* | X276_21465 | operon_0757 | 0.55 | 0.84 | 0.83 | 0.91 | 0.95 | 1.00 | 0.98 | 0.96 | 0.89 | 0.78 | 0.92 | -0.70 |
| *fabZ* | X276_21460 | operon_0757 | 0.57 | 0.85 | 0.82 | 0.92 | 0.95 | 0.98 | 1.00 | 0.97 | 0.89 | 0.76 | 0.91 | -0.59 |
| *accC* | X276_21455 | operon_0757 | 0.61 | 0.87 | 0.87 | 0.93 | 0.97 | 0.96 | 0.97 | 1.00 | 0.96 | 0.86 | 0.92 | -0.63 |
| *accD* | X276_21450 | operon_0757 | 0.72 | 0.89 | 0.93 | 0.93 | 0.94 | 0.89 | 0.89 | 0.96 | 1.00 | 0.97 | 0.89 | -0.70 |
| *accA* | X276_21445 | operon_0757 | 0.77 | 0.87 | 0.94 | 0.89 | 0.88 | 0.78 | 0.76 | 0.86 | 0.97 | 1.00 | 0.80 | -0.77 |
| *acp* | X276_20685 | operon_0820 | 0.50 | 0.78 | 0.81 | 0.84 | 0.87 | 0.92 | 0.91 | 0.92 | 0.89 | 0.80 | 1.00 | -0.65 |
| *cfa* | X276_00620 | operon_3203 | -0.71 | -0.77 | -0.80 | -0.74 | -0.72 | -0.70 | -0.59 | -0.63 | -0.70 | -0.77 | -0.65 | 1.00 |

| **Table A7: Phospholipid synthesis - RPKM values** | | | | | | |  |  |  |  |  |  |  |  |  |
| --- | --- | --- | --- | --- | --- | --- | --- | --- | --- | --- | --- | --- | --- | --- | --- |
|  |  |  |  | Shocked ABE fermentation | | | | | | Standard ABE fermentation | | | | | |
|  | Gene | Putative physiological role | Locus tag | T_b_0 | T_b_1 | T_b_2 | T_b_3 | T_b_4 | T_b_5 | T1 | T2 | T3 | T4 | T5 | T6 |
|  | **CDP - DAG synthesis** | |  |  |  |  |  |  |  |  |  |  |  |  |  |
|  | *dgk* | diacylglycerolkinase | X276_22500 | 116.25 | 122.47 | 138.05 | 141.48 | 132.66 | 120.71 | 108.25 | 119.18 | 172.54 | 182.16 | 108.36 | 79.58 |
|  | *plsX* | phosphate acyltransferase | X276_20690 | 105.64 | 84.62 | 99.32 | 113.58 | 117.31 | 102.57 | 52.48 | 93.94 | 84.86 | 154.72 | 164.52 | 97.80 |
|  | *plsY* | glycerol-3-phosphate acyltransferase | X276_06725 | 9.97 | 11.52 | 10.20 | 15.17 | 17.24 | 14.14 | 12.79 | 11.73 | 13.84 | 21.59 | 9.27 | 10.73 |
|  | *acpP* | acyl-carrier protein | X276_20685 | 1330.24 | 1063.80 | 1034.85 | 1361.60 | 1413.45 | 1424.45 | 2619.15 | 1787.95 | 2186.85 | 3282.32 | 2091.24 | 1582.81 |
|  | *plsC* | 1-acylglycerol-3-phosphate O-acyltransferase | X276_00470 | 97.46 | 63.29 | 86.82 | 100.03 | 94.42 | 100.67 | 70.67 | 106.76 | 84.14 | 104.63 | 97.44 | 74.64 |
|  | *cds* | CDP- diacylglycerol synthase | X276_20555 | 110.27 | 92.74 | 102.31 | 122.12 | 123.09 | 118.62 | 72.44 | 101.87 | 96.98 | 101.56 | 107.45 | 78.60 |
|  | **PS, PE, PC synthesis** | |  |  |  |  |  |  |  |  |  |  |  |  |  |
|  | *pssA* | phosphatidylserine synthase (type II) | X276_23850 | 176.22 | 55.68 | 73.36 | 101.19 | 129.45 | 152.82 | 224.47 | 176.64 | 198.89 | 382.76 | 240.84 | 161.30 |
|  | *psd* | phosphatidylserine decarboxylase | X276_26685 | 106.66 | 119.86 | 127.15 | 144.50 | 115.11 | 134.34 | 129.86 | 124.73 | 106.93 | 111.89 | 89.25 | 76.16 |
|  | *pmt* | phosphoethanolamine N-methyltransferase | X276_12160 | 27.44 | 22.85 | 27.36 | 29.16 | 27.93 | 34.54 | 19.78 | 33.86 | 30.81 | 34.21 | 24.68 | 23.70 |
|  | **PG synthesis** | |  |  |  |  |  |  |  |  |  |  |  |  |  |
|  | *pgsA* | phosphatidylglycerolphosphate synthase | X276_20445 | 315.35 | 209.06 | 261.34 | 314.82 | 380.64 | 369.51 | 242.91 | 313.53 | 412.56 | 497.77 | 409.95 | 308.07 |
|  | *pgp* | phosphatidylglycerophosphatase | X276_14965 | 16.64 | 16.00 | 16.84 | 14.45 | 27.96 | 20.51 | 5.18 | 14.70 | 14.91 | 14.27 | 14.91 | 13.28 |
|  | **LPG synthesis** | |  |  |  |  |  |  |  |  |  |  |  |  |  |
|  | *mprF* | phosphatidylglycerol lysyltransferase | X276_04955 | 3.15 | 4.58 | 5.00 | 5.26 | 5.89 | 5.46 | 4.47 | 5.04 | 5.19 | 8.72 | 14.11 | 14.21 |
|  | **CL synthesis** | |  |  |  |  |  |  |  |  |  |  |  |  |  |
|  | *clsA* | cardiolipin synthase | X276_02880 | 42.50 | 39.02 | 49.80 | 52.13 | 45.68 | 41.95 | 50.83 | 41.69 | 40.34 | 53.86 | 118.39 | 159.51 |

| **Table A8: Tet/AcrR family regulators and efflux pumps activation - RPKM values** | | | | | | | | | | |  |  |  |  |
| --- | --- | --- | --- | --- | --- | --- | --- | --- | --- | --- | --- | --- | --- | --- |
|  |  |  | Shocked ABE fermentation | | | | | | Standard ABE fermentation | | | | | |
| Gene | Putative physiological role | Locus tag | T_b_0 | T_b_1 | T_b_2 | T_b_3 | T_b_4 | T_b_5 | T1 | T2 | T3 | T4 | T5 | T6 |
| *tet/acrR* | Tet/AcrR transcriptional regulator | X276_20245 | 87.89 | 1322.38 | 1126.17 | 669.02 | 474.76 | 366.54 | 302.16 | 163.79 | 56.35 | 46.52 | 63.10 | 129.13 |
| *epc* | endopeptidase | X276_20240 | 143.57 | 2715.83 | 2204.12 | 1364.46 | 895.76 | 660.55 | 229.52 | 265.67 | 104.91 | 52.84 | 109.18 | 445.00 |
| *mate* | multidrug efflux protein | X276_20235 | 11.94 | 119.96 | 148.12 | 102.48 | 60.37 | 38.95 | 6.36 | 15.82 | 5.86 | 7.93 | 8.54 | 11.20 |
| *hth* | helix-turn-helix transcriptional regulator | X276_20230 | 39.09 | 106.16 | 134.94 | 106.98 | 80.82 | 59.10 | 25.37 | 49.36 | 41.63 | 44.20 | 44.17 | 37.70 |
|  |  |  |  |  |  |  |  |  |  |  |  |  |  |  |
| *tet/acrR* | Tet/AcrR transcriptional regulator | X276_19550 | 31.45 | 247.69 | 390.40 | 341.49 | 238.44 | 232.69 | 23.72 | 43.21 | 51.58 | 63.84 | 97.07 | 290.91 |
| *mfs* | DHA2 family efflux MFS transporter permease subunit | X276_19545 | 1.74 | 41.63 | 69.04 | 40.47 | 19.48 | 18.02 | 1.45 | 2.74 | 3.52 | 5.39 | 12.30 | 49.31 |
| *mem* | membrane protein | X276_19540 | 2.80 | 4.26 | 4.55 | 5.39 | 4.21 | 3.65 | 2.48 | 6.45 | 6.17 | 8.62 | 15.67 | 30.44 |
|  |  |  |  |  |  |  |  |  |  |  |  |  |  |  |
| *abc* | ABC transporter permease | X276_04725 | 423.07 | 988.72 | 881.28 | 550.75 | 368.87 | 302.45 | 721.25 | 543.60 | 168.21 | 253.69 | 151.09 | 126.03 |
| *abc* | ABC transporter permease | X276_04720 | 577.85 | 1530.24 | 1386.19 | 1006.10 | 696.42 | 555.65 | 754.02 | 624.18 | 198.41 | 291.82 | 191.77 | 183.22 |
| *abc* | ABC transporter ATP-binding protein | X276_04715 | 503.29 | 1362.12 | 1133.50 | 760.71 | 535.90 | 456.39 | 697.44 | 542.90 | 159.28 | 240.69 | 171.18 | 173.10 |
| *tet/acrR* | Tet/AcrR transcriptional regulator | X276_04710 | 657.16 | 1112.03 | 1082.13 | 933.59 | 687.44 | 559.63 | 805.97 | 592.87 | 227.49 | 318.45 | 246.20 | 227.46 |
|  |  |  |  |  |  |  |  |  |  |  |  |  |  |  |
| *abc* | ABC transporter ATP-binding protein | X276_06630 | 1.25 | 3.96 | 2.41 | 2.71 | 2.73 | 3.86 | 1.36 | 1.71 | 1.59 | 3.02 | 3.90 | 3.15 |
| *abc* | ABC transporter ATP-binding protein | X276_06625 | 1.00 | 2.25 | 2.23 | 2.56 | 2.94 | 1.11 | 0.78 | 1.13 | 1.20 | 1.54 | 1.28 | 1.40 |
| *marR* | MarR family transcriptional regulator | X276_06620 | 21.51 | 31.89 | 26.68 | 40.70 | 24.63 | 24.73 | 15.36 | 24.50 | 22.96 | 20.03 | 24.63 | 19.96 |
| *tet/acrR* | Tet/AcrR transcriptional regulator | X276_06615 | 6.37 | 17.37 | 11.37 | 16.45 | 12.26 | 7.91 | 5.32 | 6.12 | 5.76 | 4.39 | 5.37 | 4.85 |
|  |  |  |  |  |  |  |  |  |  |  |  |  |  |  |
| *marR* | MarR family transcriptional regulator | X276_07835 | 6.86 | 35.00 | 37.91 | 27.73 | 24.99 | 18.76 | 10.31 | 12.32 | 15.57 | 19.66 | 16.36 | 17.69 |
| *tet/acrR* | Tet/AcrR transcriptional regulator | X276_07830 | 40.36 | 55.27 | 51.49 | 53.97 | 60.91 | 56.36 | 42.53 | 53.78 | 59.85 | 64.48 | 62.26 | 70.59 |
| *mfs* | multidrug efflux MFS transporter | X276_07825 | 4.82 | 7.54 | 5.63 | 7.58 | 4.37 | 8.28 | 5.36 | 6.60 | 4.29 | 10.70 | 14.05 | 5.83 |
|  |  |  |  |  |  |  |  |  |  |  |  |  |  |  |
| *mfs* | multidrug efflux MFS transporter | X276_09550 | 13.69 | 45.45 | 58.25 | 77.10 | 67.46 | 70.41 | 7.95 | 21.07 | 19.38 | 30.58 | 38.74 | 45.13 |
| *tet/acrR* | Tet/AcrR transcriptional regulator | X276_09545 | 51.06 | 59.76 | 70.50 | 83.52 | 68.01 | 69.98 | 51.73 | 68.65 | 80.45 | 57.21 | 36.48 | 41.80 |
|  |  |  |  |  |  |  |  |  |  |  |  |  |  |  |
| *mfs* | multidrug efflux MFS transporter | X276_12180 | 3.45 | 15.78 | 19.10 | 21.72 | 21.25 | 22.57 | 1.63 | 4.23 | 3.98 | 4.38 | 7.19 | 7.25 |
| *tet/acrR* | Tet/AcrR transcriptional regulator | X276_12175 | 1.91 | 2.36 | 5.49 | 5.07 | 4.64 | 2.63 | 0.55 | 1.84 | 1.53 | 3.43 | 2.45 | 2.41 |
|  |  |  |  |  |  |  |  |  |  |  |  |  |  |  |
| *tet/acrR* | Tet/AcrR transcriptional regulator | X276_14280 | 159.13 | 45.99 | 45.68 | 51.78 | 66.63 | 72.72 | 81.42 | 135.37 | 95.99 | 53.90 | 27.01 | 28.96 |
| *rnd* | efflux RND transporter periplasmic adaptor | X276_14275 | 178.13 | 53.08 | 55.83 | 70.74 | 85.23 | 81.70 | 95.88 | 184.28 | 130.80 | 92.16 | 40.10 | 37.12 |
| *rnd* | efflux RND transporter permease subunit | X276_14270 | 132.88 | 54.99 | 57.92 | 65.11 | 75.12 | 68.97 | 103.16 | 135.93 | 97.00 | 76.56 | 44.52 | 47.25 |
| *tolC* | TolC family protein | X276_14265 | 413.48 | 318.81 | 300.92 | 333.86 | 308.49 | 371.38 | 415.65 | 488.00 | 425.15 | 263.14 | 172.19 | 164.45 |
| *tet/acrR* | TetR/AcrR transcriptional regulator | X276_14260 | 21.13 | 12.93 | 20.62 | 15.81 | 25.31 | 26.30 | 26.47 | 22.06 | 27.37 | 43.26 | 27.97 | 22.76 |
|  |  |  |  |  |  |  |  |  |  |  |  |  |  |  |
| **Table A8 additional information: Tet/AcrR family regulators and efflux pumps activation - Putative operon organization** | | | | | | | | | | | | | | |
|  |  | Genome2D |  | Correlation matrix | | | | |  |  |  |  |  |  |
| Gene | Locus tag |  |  | _20245 | _20240 | _20235 | _20230 |  |  |  |  |  |  |  |
| *tet/acrR* | X276_20245 | operon_0865 |  | 1.00 | 0.99 | 0.95 | 0.92 |  |  |  |  |  |  |  |
| *epc* | X276_20240 | operon_0865 |  | 0.99 | 1.00 | 0.96 | 0.93 |  |  |  |  |  |  |  |
| *mate* | X276_20235 | operon_0866 |  | 0.95 | 0.96 | 1.00 | 1.00 |  |  |  |  |  |  |  |
| *hth* | X276_20230 | operon_0867 |  | 0.92 | 0.93 | 1.00 | 1.00 |  |  |  |  |  |  |  |
|  |  |  |  |  |  |  |  |  |  |  |  |  |  |  |
|  |  |  |  | _19550 | _19545 | _19540 |  |  |  |  |  |  |  |  |
| *tet/acrR* | X276_19550 | operon_0944 |  | 1.00 | 0.95 | 0.20 |  |  |  |  |  |  |  |  |
| *mfs* | X276_19545 | operon_0944 |  | 0.95 | 1.00 | 0.29 |  |  |  |  |  |  |  |  |
| *mem* | X276_19540 | operon_0945 |  | 0.20 | 0.29 | 1.00 |  |  |  |  |  |  |  |  |
|  |  |  |  |  |  |  |  |  |  |  |  |  |  |  |
|  |  |  |  | _04725 | _04720 | _04715 | _04710 |  |  |  |  |  |  |  |
| *abc* | X276_04725 | operon_2751 |  | 1.00 | 0.96 | 0.98 | 0.96 |  |  |  |  |  |  |  |
| *abc* | X276_04720 | operon_2751 |  | 0.96 | 1.00 | 0.99 | 0.98 |  |  |  |  |  |  |  |
| *abc* | X276_04715 | operon_2751 |  | 0.98 | 0.99 | 1.00 | 0.97 |  |  |  |  |  |  |  |
| *tet/acrR* | X276_04710 | operon_2751 |  | 0.96 | 0.98 | 0.97 | 1.00 |  |  |  |  |  |  |  |
|  |  |  |  |  |  |  |  |  |  |  |  |  |  |  |
|  |  |  |  | _06630 | _06625 | _06620 | _06615 |  |  |  |  |  |  |  |
| *abc* | X276_06630 | operon_2527 |  | 1.00 | 0.47 | 0.49 | 0.55 |  |  |  |  |  |  |  |
| *abc* | X276_06625 | operon_2527 |  | 0.47 | 1.00 | 0.76 | 0.87 |  |  |  |  |  |  |  |
| *marR* | X276_06620 | operon_2528 |  | 0.49 | 0.76 | 1.00 | 0.92 |  |  |  |  |  |  |  |
| *tet/acrR* | X276_06615 | operon_2529 |  | 0.55 | 0.87 | 0.92 | 1.00 |  |  |  |  |  |  |  |
|  |  |  |  |  |  |  |  |  |  |  |  |  |  |  |
|  |  |  |  | _07835 | _07830 | _07825 |  |  |  |  |  |  |  |  |
| *marR* | X276_07835 | operon_2385 |  | 1.00 | 0.56 | 0.15 |  |  |  |  |  |  |  |  |
| *tet/acrR* | X276_07830 | operon_2386 |  | 0.56 | 1.00 | 0.14 |  |  |  |  |  |  |  |  |
| *mfs* | X276_07825 | operon_2387 |  | 0.15 | 0.14 | 1.00 |  |  |  |  |  |  |  |  |
|  |  |  |  |  |  |  |  |  |  |  |  |  |  |  |
|  |  |  |  | _09550 | _09545 |  |  |  |  |  |  |  |  |  |
| *mfs* | X276_09550 | operon_2167 |  | 1.00 | 0.42 |  |  |  |  |  |  |  |  |  |
| *tet/acrR* | X276_09545 | operon_2168 |  | 0.42 | 1.00 |  |  |  |  |  |  |  |  |  |
|  |  |  |  |  |  |  |  |  |  |  |  |  |  |  |
|  |  |  |  | _12180 | _12175 |  |  |  |  |  |  |  |  |  |
| *mfs* | X276_12180 | operon_1823 |  | 1.00 | 0.80 |  |  |  |  |  |  |  |  |  |
| *tet/acrR* | X276_12175 | operon_1824 |  | 0.80 | 1.00 |  |  |  |  |  |  |  |  |  |
|  |  |  |  |  |  |  |  |  |  |  |  |  |  |  |
|  |  |  |  | _14280 | _14275 | _14270 | _14265 | _14260 |  |  |  |  |  |  |
| *tet/acrR* | X276_14280 | operon_1585 |  | 1.00 | 0.98 | 0.96 | 0.79 | -0.04 |  |  |  |  |  |  |
| *rnd* | X276_14275 | operon_1585 |  | 0.98 | 1.00 | 0.95 | 0.77 | 0.01 |  |  |  |  |  |  |
| *rnd* | X276_14270 | operon_1585 |  | 0.96 | 0.95 | 1.00 | 0.86 | 0.08 |  |  |  |  |  |  |
| *tolC* | X276_14265 | operon_1586 |  | 0.79 | 0.77 | 0.86 | 1.00 | -0.15 |  |  |  |  |  |  |
| *tet/acrR* | X276_14260 | operon_1587 |  | -0.04 | 0.01 | 0.08 | -0.15 | 1.00 |  |  |  |  |  |  |
